# Supplementary material for: Transcription-mediated organization of the replication initiation program across large genes sets common fragile sites genome-wide
Source: Nat Commun. 2019 Dec 13;10:5693. doi: 10.1038/s41467-019-13674-5 (PMC6911102; doi:10.1038/s41467-019-13674-5)
Supplement: Supplementary file 1 — Supplementary Information [file 41467_2019_13674_MOESM1_ESM.pdf]

SUPPLEMENTARY INFORMATION

Transcription-mediated Organization of the Replication Initiation Program Across Large Genes Sets Common Fragile Sites Genome-wide

Brison et al.

Supplementary Figure 1

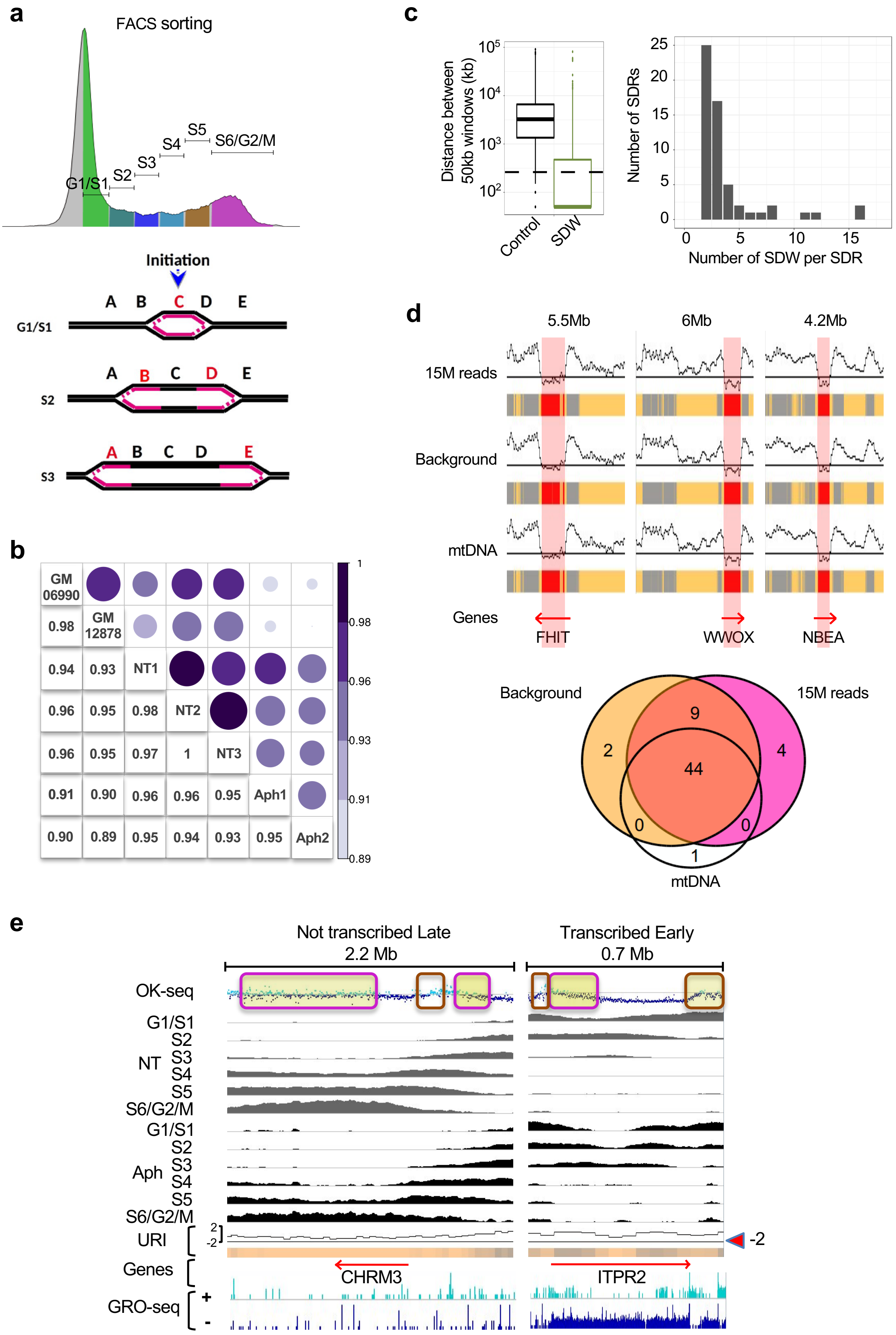

Supplementary Figure 1. Repli-Seq analysis of lymphoblasts treated or not with aphidicolin. **(a)** Scheme of Repli-Seq experiments. Upper panel: Cells at six successive periods of S phase (G1/S1, S2, S3, S4, S5 and S6/G2/M) were obtained by FACS sorting from asynchronous cell populations previously pulse labelled with BrdU. Lower panel: Schematic representation of forks progressing from an early-firing initiation zone. Evolving position of BrdU-labelled DNA (red) is shown across the replicon at the different periods of S phase. Purification and sequencing of BrdU-labelled DNA at the six steps of the S phase allow the genome-wide reconstitution of replication dynamics. **(b)** Correlation coefficients (Pearson's *r*) of replication timing (S50) between untreated GM06990 and GM12878 lymphoblastoid cells, untreated JEFF cells (3 replicates, NT1, NT2 and NT3) and JEFF cells treated with Aph (2 replicates, Aph1 and Aph2). Colour intensity and the size of the circle are proportional to the correlation coefficients. **(c)** Clustering of SDW. Left panel: Boxplots comparing the distance separating SDWs (n=330) upon random simulation (Control, black) or upon analysis of our data (green). SDWs separated by less than 250 kb were merged as SDR (Methods). Right panel: Histogram showing the distribution of SDRs according to their SDW content. **(d)** Comparison of different normalisation methods. Upper panel: URI profiles (as in Fig. 1f) at three loci upon normalization to the same total number of reads (i.e 15 M), by adjusting the background to the same level (background) or by the level of mitochondrial DNA (mtDNA). Lower panel: Veen diagram showing the number of overlapping SDRs with the three normalization methods. **(e)** Representative examples of large genes without T-SDR/SDW (displayed as in Fig. 2c): Late-replicating and not (or very weakly) transcribed (Left), early-replicating and transcribed (right). The genomic regions displayed are from left to right: chr1:238.7-240.9 Mb; and chr12:26.4-27.1 Mb. Source data are provided as a Source Data file.

Supplementary Figure 2

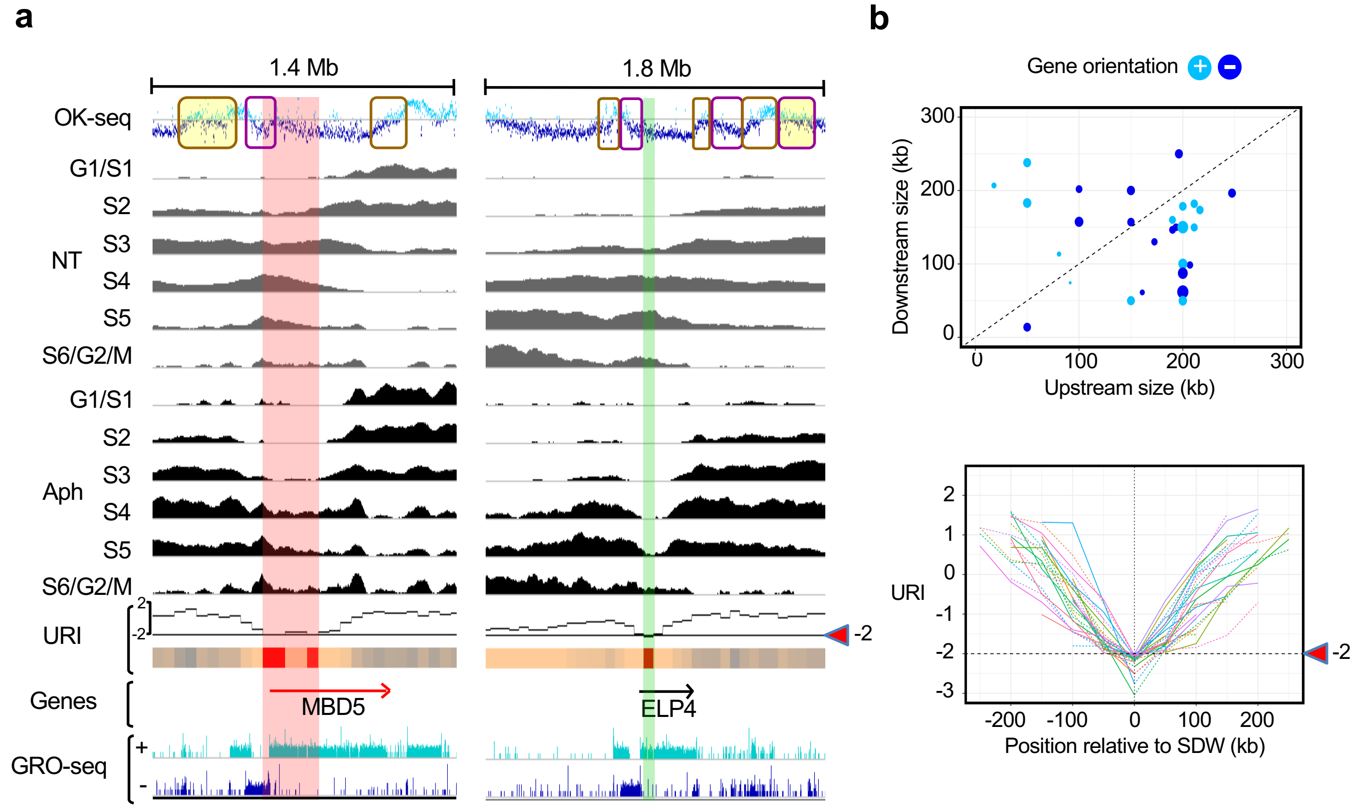

Supplementary Figure 2. Relationships between replication forks and transcription directionality (a) Asymmetric termination zones lying in the 5' end of two large genes in untreated cells, so that forks progress head-on with transcription all along the gene in untreated cells and across most of the gene in Aph-treated cells. Ok-Seq, Repli-Seq, URI and GRO-Seq profiles are shown as in Fig. 2c. The displayed genomic regions are chr2:148.2-149.6 Mb and chr11:30.7-32.5 Mb, from left to right. **(b)** Kinetics of URI decrease along sequences flanking the T-SDWs (n=45, upper and lower panels: as in Fig. 2e). Source data are provided as a Source Data file.

Supplementary Figure 3

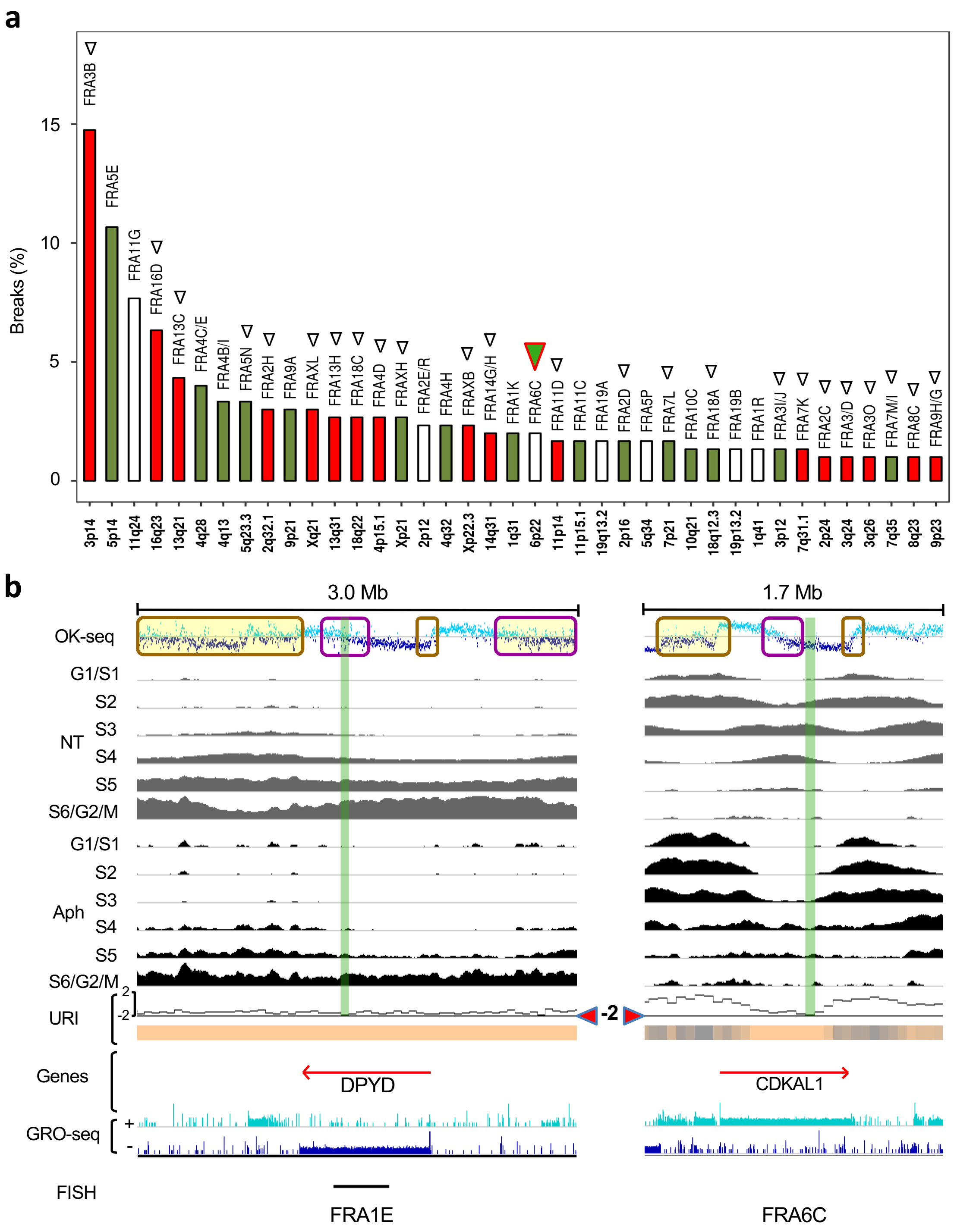

Supplementary Figure 3. Correlations between CFSs mapped by conventional cytogenetics and SDRs/SDWs (a) The 39 CFSs with a break frequency ≥1% mapped in JEFF cells are presented according to Mraseck *et al.* [32] and ordered by decreasing break frequency. Coloured bands contain at least one SDR (red) and/or SDW (green). CFSs identified by a white arrowhead are associated with T-SDRs or T-SDWs. FRA6C/H (green arrowhead) might be a false negative (see Supplementary Fig. 3b). **(b)** Examples of expressed large genes instable upon Aph treatment but free of T-SDRs/SDWs that might be false negative. OK-Seq, Repli-Seq, URI and GRO-Seq profiles are presented as in Fig. 2c. Both genes are free of SDR/SDW, but the URI profiles show delayed regions close to -2, all along the very late domain hosting the gene (*DPYD*), or in the middle of the gene (*CDKAL1*), like in canonical CFSs. Green vertical bars point to the most delayed windows, i.e. putative SDWs. The genomic regions displayed are chr1:96.4-99.4 Mb and chr6:20.1-21.8 Mb. Source data are provided as a Source Data file.

Supplementary Figure 4

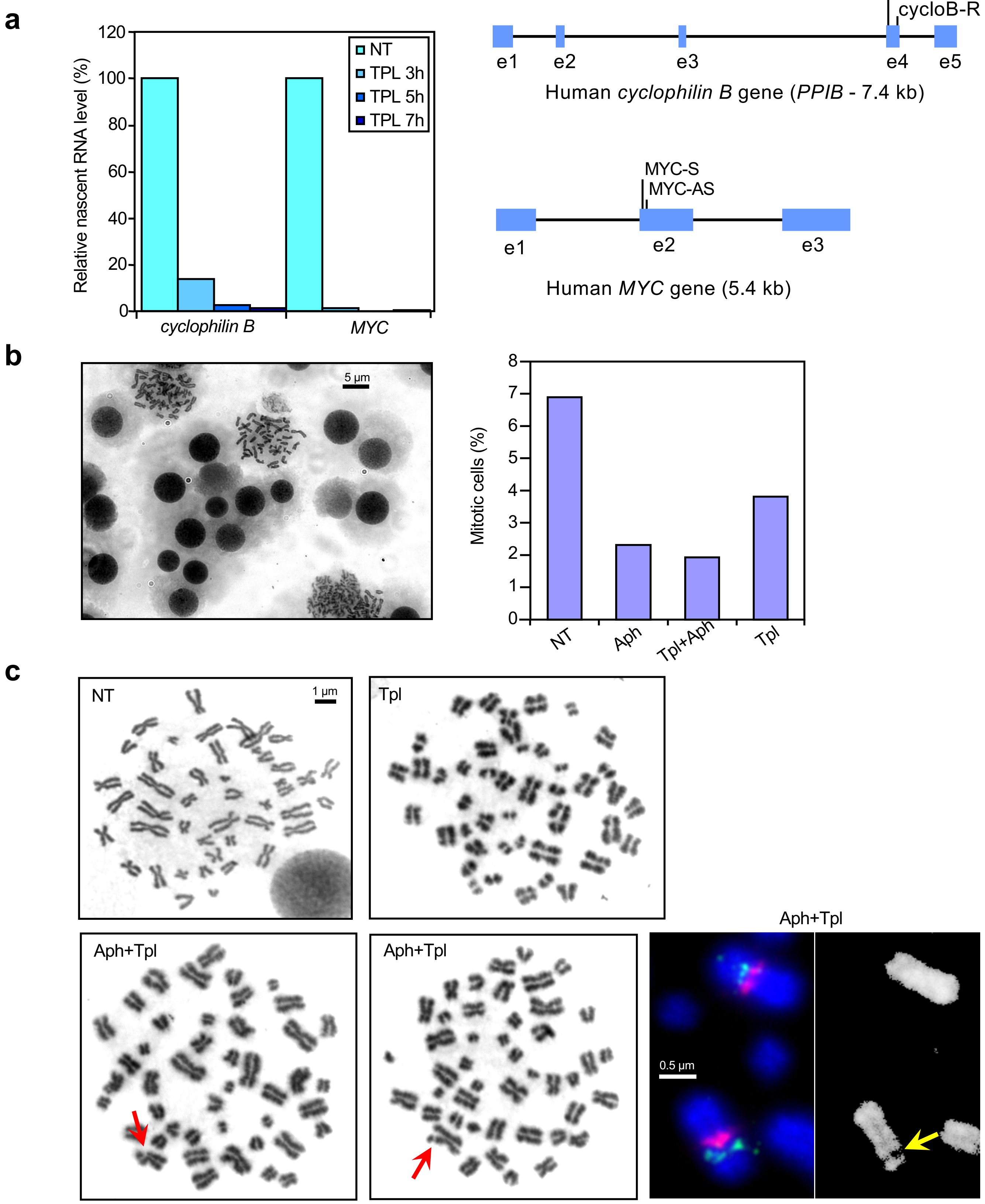

Supplementary Figure 4. Impact of Tpl on transcription, cell cycle progression and CFS instability **(a)** Maps of the cyclophilin B (*PPIB*) and *MYC* genes with the positions of exons and primer pairs (right panel) used to quantify nascent RNAs by RT-qPCR in the indicated conditions (left panel) are shown as in Fig. 4a. **(b)** Microscopic counting of metaphase plates relative to the total number of nuclei after Giemsa staining (left panel) allows determination of the mitotic index in each condition (right panel). **(c)** Representative examples of Giemsa stained metaphase plates from untreated cells (NT), cells treated with Tpl alone or with Tpl+Aph (the red arrow points to a chromosome break). Note that the chromosomes of Tpl-treated cells are over-condensed. Example of break at FRA3B (yellow arrow) visualized after DAPI staining and FISH with probes specific to *FHIT* (green) and to the centromere of chromosome 3 (red) after Tpl+Aph treatment. Contrast of DAPI stained chromosomes (right panels) was enhanced to clearly show the break (compare to the unbroken second allele). Source data are provided as a Source Data file.
