# Supplementary material for: Transcription-mediated organization of the replication initiation program across large genes sets common fragile sites genome-wide
Source: Nat Commun. 2019 Dec 13;10:5693. doi: 10.1038/s41467-019-13674-5 (PMC6911102; doi:10.1038/s41467-019-13674-5)
Supplement: Supplementary file 3 — Description of Additional Supplementary Files [file 41467_2019_13674_MOESM3_ESM.pdf]

Description of Additional Supplementary Files

File Name: Supplementary Data 1
Description: Significantly delayed region (SDR) characterization.

File Name: Supplementary Data 2
Description: Significantly delayed window (SDW) characterization.

File Name: Supplementary Data 3
Description: CFSs mapped by conventional cytogenetics.
