# Supplementary material for: Transcription-mediated organization of the replication initiation program across large genes sets common fragile sites genome-wide
Source: Nat Commun. 2019 Dec 13;10:5693. doi: 10.1038/s41467-019-13674-5 (PMC6911102; doi:10.1038/s41467-019-13674-5)
Supplement: Supplementary file 7 — Reporting Summary [file 41467_2019_13674_MOESM7_ESM.pdf]

nature research

Corresponding author(s): C.L. Chen, M. Debatisse

Last updated by author(s): Nov 3, 2019

# Reporting Summary

Nature Research wishes to improve the reproducibility of the work that we publish. This form provides structure for consistency and transparency in reporting. For further information on Nature Research policies, see Authors & Referees and the Editorial Policy Checklist.

## Statistics

For all statistical analyses, confirm that the following items are present in the figure legend, table legend, main text, or Methods section.

| n/a | Confirmed | |
|---|---|---|
| ☐ | ☒ | The exact sample size (*n*) for each experimental group/condition, given as a discrete number and unit of measurement |
| ☐ | ☒ | A statement on whether measurements were taken from distinct samples or whether the same sample was measured repeatedly |
| ☐ | ☒ | The statistical test(s) used AND whether they are one- or two-sided<br>*Only common tests should be described solely by name; describe more complex techniques in the Methods section.* |
| ☒ | ☐ | A description of all covariates tested |
| ☐ | ☒ | A description of any assumptions or corrections, such as tests of normality and adjustment for multiple comparisons |
| ☐ | ☒ | A full description of the statistical parameters including central tendency (e.g. means) or other basic estimates (e.g. regression coefficient) AND variation (e.g. standard deviation) or associated estimates of uncertainty (e.g. confidence intervals) |
| ☐ | ☒ | For null hypothesis testing, the test statistic (e.g. *F*, *t*, *r*) with confidence intervals, effect sizes, degrees of freedom and *P* value noted<br>*Give P values as exact values whenever suitable.* |
| ☒ | ☐ | For Bayesian analysis, information on the choice of priors and Markov chain Monte Carlo settings |
| ☒ | ☐ | For hierarchical and complex designs, identification of the appropriate level for tests and full reporting of outcomes |
| ☒ | ☐ | Estimates of effect sizes (e.g. Cohen's *d*, Pearson's *r*), indicating how they were calculated |

*Our web collection on statistics for biologists contains articles on many of the points above.*

## Software and code

Policy information about availability of computer code

| | |
|---|---|
| Data collection | The data used in current study have been either generated for the purpose of the study or downloaded directly from GEO, no additional software or code was used to collect the data. |
| Data analysis | The sequencing data were demultiplexed using the distribution of CASAVA software (CASAVA-1.8.2 bcl2fastq2 v2.18.12). Illumina adapters were removed using Cutadapt-1.15. The reads were mapped on the reference using bwa-0.6.2-r126. The PCR duplicates were removed by Picard tools (http://broadinstitute.github.io/picard). The Repli-Seq, Ok-Seq, Bubble-Seq and GRO-Seq data were analyzed using Python (v2.7.9) and R (v3.4.4). The computer codes and further processing data are available on the GitHub repositories of the team (https://github.com/CL-CHEN-Lab/). Additional codes are available upon request. |

For manuscripts utilizing custom algorithms or software that are central to the research but not yet described in published literature, software must be made available to editors/reviewers. We strongly encourage code deposition in a community repository (e.g. GitHub). See the Nature Research guidelines for submitting code & software for further information.

## Data

Policy information about availability of data

All manuscripts must include a data availability statement. This statement should provide the following information, where applicable:

- Accession codes, unique identifiers, or web links for publicly available datasets
- A list of figures that have associated raw data
- A description of any restrictions on data availability

The GEO accession number of the data produced in this manuscript is GSE134709. https://www.ncbi.nlm.nih.gov/geo/query/acc.cgi?acc= GSE134709.

# Field-specific reporting

Please select the one below that is the best fit for your research. If you are not sure, read the appropriate sections before making your selection.

☒ Life sciences ☐ Behavioural & social sciences ☐ Ecological, evolutionary & environmental sciences

For a reference copy of the document with all sections, see nature.com/documents/nr-reporting-summary-flat.pdf

# Life sciences study design

All studies must disclose on these points even when the disclosure is negative.

| | |
|---|---|
| Sample size | 3 biological replicates of Repli-Seq experiments of human lymphoblastoid cells grown in the absence of aphidicolin and 2 biological replicates in the presence (600 nM, 16h, 2 biological replicates) of aphidicolin. |
| Data exclusions | When a region was not significantly enriched in all 6 periods of S phase samples, no replication timing value was computed (~5% of genome regions, most located close to telomeres or centromeres). |
| Replication | The Repli-Seq profiles were highly reproducible between 3 biological replicates of human lymphoblastoid JEFF cells grown under normal conditions, as well as between the 2 biological replicates for JEFF cells treated with aphidicolin. |
| Randomization | Randomization is not relevant to our study as we aim to compare the replication dynamics of cells with or without aphidicolin treatment. |
| Blinding | Blinding is not relevant to our study as we aim to compare the replication dynamics of cells with or without aphidicolin treatment. |

# Reporting for specific materials, systems and methods

We require information from authors about some types of materials, experimental systems and methods used in many studies. Here, indicate whether each material, system or method listed is relevant to your study. If you are not sure if a list item applies to your research, read the appropriate section before selecting a response.

## Materials & experimental systems

| n/a | Involved in the study | |
|---|---|---|
| ☐ | ☒ | Antibodies |
| ☐ | ☒ | Eukaryotic cell lines |
| ☒ | ☐ | Palaeontology |
| ☒ | ☐ | Animals and other organisms |
| ☒ | ☐ | Human research participants |
| ☒ | ☐ | Clinical data |

## Methods

| n/a | Involved in the study | |
|---|---|---|
| ☐ | ☒ | ChIP-seq |
| ☐ | ☒ | Flow cytometry |
| ☒ | ☐ | MRI-based neuroimaging |

## Antibodies

| | |
|---|---|
| Antibodies used | anti-BrdU monoclonal antibody (BD Biosciences, 347580) |
| Validation | Specificity checked by the supplier. |

## Eukaryotic cell lines

Policy information about cell lines

| | |
|---|---|
| Cell line source(s) | JEFF cells (established from human B-lymphocytes of a healthy individual. |
| Authentication | none |
| Mycoplasma contamination | The cell cultures were regularly tested and no mycoplasma contamination was found. |
| Commonly misidentified lines (See ICLAC register) | *Name any commonly misidentified cell lines used in the study and provide a rationale for their use.* |

## ChIP-seq

### Data deposition

☒ Confirm that both raw and final processed data have been deposited in a public database such as GEO.

☐ Confirm that you have deposited or provided access to graph files (e.g. BED files) for the called peaks.

| | |
|---|---|
| Data access links<br>*May remain private before publication.* | The GEO accession number of the data produced in this manuscript is GSE134709. |
| Files in database submission | Files in database submission:<br>- processed data file<br>NT.Rep1.S1G1.bw<br>NT.Rep1.S2.bw<br>NT.Rep1.S3.bw<br>NT.Rep1.S4.bw<br>NT.Rep1.S5.bw<br>NT.Rep1.S6G2M.bw<br>NT.Rep2.S1G1.bw<br>NT.Rep2.S2.bw<br>NT.Rep2.S3.bw<br>NT.Rep2.S4.bw<br>NT.Rep2.S5.bw<br>NT.Rep2.S6G2M.bw<br>NT.Rep3.S1G1.bw<br>NT.Rep3.S2.bw<br>NT.Rep3.S3.bw<br>NT.Rep3.S4.bw<br>NT.Rep3.S5.bw<br>NT.Rep3.S6G2M.bw<br>Aph.Rep1.S1G1.bw<br>Aph.Rep1.S2.bw<br>Aph.Rep1.S3.bw<br>Aph.Rep1.S4.bw<br>Aph.Rep1.S5.bw<br>Aph.Rep1.S6G2M.bw<br>Aph.Rep2.S1G1.bw<br>Aph.Rep2.S2.bw<br>Aph.Rep2.S3.bw<br>Aph.Rep2.S4.bw<br>Aph.Rep2.S5.bw<br>Aph.Rep2.S6G2M.bw<br>NT.S1G1.bw<br>NT.S2.bw<br>NT.S3.bw<br>NT.S4.bw<br>NT.S5.bw<br>NT.S6G2M.bw<br>Aph.S1G1.bw<br>Aph.S2.bw<br>Aph.S3.bw<br>Aph.S4.bw<br>Aph.S5.bw<br>Aph.S6G2M.bw<br>Replication_Index.bw<br><br>- Raw sequencing data file<br>NT.Rep1.S1G1.R1.fastq.gz<br>NT.Rep1.S1G1.R2.fastq.gz<br>NT.Rep1.S2.R1.fastq.gz<br>NT.Rep1.S2.R2.fastq.gz<br>NT.Rep1.S3.R1.fastq.gz<br>NT.Rep1.S3.R2.fastq.gz<br>NT.Rep1.S4.R1.fastq.gz<br>NT.Rep1.S4.R2.fastq.gz<br>NT.Rep1.S5.R1.fastq.gz<br>NT.Rep1.S5.R2.fastq.gz<br>NT.Rep1.S6G2M.R1.fastq.gz<br>NT.Rep1.S6G2M.R2.fastq.gz<br><br>NT.Rep2.S1G1.Run1.R1.fastq.gz<br>NT.Rep2.S1G1.Run1.R2.fastq.gz |

NT.Rep2.S2.Run1.R1.fastq.gz
NT.Rep2.S2.Run1.R2.fastq.gz
NT.Rep2.S3.Run1.R1.fastq.gz
NT.Rep2.S3.Run1.R2.fastq.gz
NT.Rep2.S4.Run1.R1.fastq.gz
NT.Rep2.S4.Run1.R2.fastq.gz
NT.Rep2.S5.Run1.R1.fastq.gz
NT.Rep2.S5.Run1.R2.fastq.gz
NT.Rep2.S6G2M.Run1.R1.fastq.gz
NT.Rep2.S6G2M.Run1.R2.fastq.gz

NT.Rep2.S1G1.Run2.R1.fastq.gz
NT.Rep2.S1G1.Run2.R2.fastq.gz
NT.Rep2.S2.Run2.R1.fastq.gz
NT.Rep2.S2.Run2.R2.fastq.gz
NT.Rep2.S4.Run2.R1.fastq.gz
NT.Rep2.S4.Run2.R2.fastq.gz
NT.Rep2.S6G2M.Run2.R1.fastq.gz
NT.Rep2.S6G2M.Run2.R2.fastq.gz

NT.Rep3.S1G1.R1.fastq.gz
NT.Rep3.S1G1.R2.fastq.gz
NT.Rep3.S2.R1.fastq.gz
NT.Rep3.S2.R2.fastq.gz
NT.Rep3.S3.R1.fastq.gz
NT.Rep3.S3.R2.fastq.gz
NT.Rep3.S4.R1.fastq.gz
NT.Rep3.S4.R2.fastq.gz
NT.Rep3.S5.R1.fastq.gz
NT.Rep3.S5.R2.fastq.gz
NT.Rep3.S6G2M.R1.fastq.gz
NT.Rep3.S6G2M.R2.fastq.gz

Aph.Rep1.S1G1.R1.fastq.gz
Aph.Rep1.S1G1.R2.fastq.gz
Aph.Rep1.S2.R1.fastq.gz
Aph.Rep1.S2.R2.fastq.gz
Aph.Rep1.S3.R1.fastq.gz
Aph.Rep1.S3.R2.fastq.gz
Aph.Rep1.S4.R1.fastq.gz
Aph.Rep1.S4.R2.fastq.gz
Aph.Rep1.S5.R1.fastq.gz
Aph.Rep1.S5.R2.fastq.gz
Aph.Rep1.S6G2M.R1.fastq.gz
Aph.Rep1.S6G2M.R2.fastq.gz

Aph.Rep2.S1G1.R1.fastq.gz
Aph.Rep2.S1G1.R2.fastq.gz
Aph.Rep2.S2.R1.fastq.gz
Aph.Rep2.S2.R2.fastq.gz
Aph.Rep2.S3.R1.fastq.gz
Aph.Rep2.S3.R2.fastq.gz
Aph.Rep2.S4.R1.fastq.gz
Aph.Rep2.S4.R2.fastq.gz
Aph.Rep2.S5.R1.fastq.gz
Aph.Rep2.S5.R2.fastq.gz
Aph.Rep2.S6G2M.R1.fastq.gz
Aph.Rep2.S6G2M.R2.fastq.gz

Genome browser session
(e.g. UCSC)

The IGV session used to visualized and generated the figures in current manuscript has been included in the Source Data files and is available upon request.

## Methodology

Replicates

The Repli-Seq profiles were highly reproducible between 3 biological replicates of human lymphoblastoid JEFF cells grown under normal conditions, as well as between the 2 biological replicates for JEFF cells treated with aphidicolin.

Sequencing depth

All are paired-end sequencing data.

- NT.Rep1.S1G1
Total number of reads: 37 533 052
Uniquely mapped reads (hg19): 28 573 449
Length of reads: 2x43

- NT.Rep1.S2
Total number of reads: 43 721 356
Uniquely mapped reads (hg19): 30 534 482
Length of reads: 2x43

- NT.Rep1.S3
Total number of reads: 21 334 370
Uniquely mapped reads (hg19): 14 508 503
Length of reads: 2x43

- NT.Rep1.S4
Total number of reads: 54 812 508
Uniquely mapped reads (hg19): 40 661 135
Length of reads: 2x43

- NT.Rep1.S5
Total number of reads: 49 485 624
Uniquely mapped reads (hg19): 35 873 838
Length of reads: 2x43

- NT.Rep1.S6G2M
Total number of reads: 43 910 096
Uniquely mapped reads (hg19): 29 622 513
Length of reads: 2x43

- NT.Rep2.S1G1
Total number of reads: 66 107 820
Uniquely mapped reads (hg19): 51 304 505
Length of reads: 2x80 or 2x75

- NT.Rep2.S2
Total number of reads: 131 686 738
Uniquely mapped reads (hg19): 100 500 853
Length of reads: 2x80 or 2x75

- NT.Rep2.S3
Total number of reads: 232 861 034
Uniquely mapped reads (hg19): 157 482 983
Length of reads: 2x80

- NT.Rep2.S4
Total number of reads: 136 477 748
Uniquely mapped reads (hg19): 96 412 381
Length of reads: 2x80 or 2x75

- NT.Rep2.S5
Total number of reads: 144 336 300
Uniquely mapped reads (hg19): 90 802 963
Length of reads: 2x80

- NT.Rep2.S6G2M
Total number of reads: 130,566,258
Uniquely mapped reads (hg19): 82,987,932
Length of reads: 2x80 or 2x75

- NT.Rep3.S1G1
Total number of reads: 54 300 456
Uniquely mapped reads (hg19): 13 407 399
Length of reads: 2x43

- NT.Rep3.S2
Total number of reads: 82 373 624
Uniquely mapped reads (hg19): 37 753 956
Length of reads: 2x43

- NT.Rep3.S3
Total number of reads: 58 234 052
Uniquely mapped reads (hg19): 27 089 061
Length of reads: 2x43

- NT.Rep3.S4
Total number of reads: 82 558 022

Uniquely mapped reads (hg19): 35 637 176
Length of reads: 2x43

- NT.Rep3.S5
Total number of reads: 78 000 272
Uniquely mapped reads (hg19): 34 877 742
Length of reads: 2x43

- NT.Rep3.S6G2M
Total number of reads: 79 567 622
Uniquely mapped reads (hg19): 22 475 001
Length of reads: 2x43

- Aph.Rep1.S1G1
Total number of reads: 54 472 268
Uniquely mapped reads (hg19): 40 003 385
Length of reads: 2x43

- Aph.Rep1.S2
Total number of reads: 54 731 298
Uniquely mapped reads (hg19): 38 352 015
Length of reads: 2x43

- Aph.Rep1.S3
Total number of reads: 55 561 586
Uniquely mapped reads (hg19): 40 390 624
Length of reads: 2x43

- Aph.Rep1.S4
Total number of reads: 50 240 262
Uniquely mapped reads (hg19): 36 856 394
Length of reads: 2x43

- Aph.Rep1.S5
Total number of reads: 62 280 088
Uniquely mapped reads (hg19): 44 393 592
Length of reads: 2x43

- Aph.Rep1.S6G2M
Total number of reads: 64 731 844
Uniquely mapped reads (hg19): 48 194 003
Length of reads: 2x43

- Aph.Rep2.S1G1
Total number of reads: 32 321 106
Uniquely mapped reads (hg19): 16 978 197
Length of reads: 2x43

- Aph.Rep2.S2
Total number of reads: 46 068 778
Uniquely mapped reads (hg19): 28 380 435
Length of reads: 2x43

- Aph.Rep2.S3
Total number of reads: 31 915 626
Uniquely mapped reads (hg19): 18 067 233
Length of reads: 2x43

- Aph.Rep2.S4
Total number of reads: 63 377 628
Uniquely mapped reads (hg19): 35 833 426
Length of reads: 2x43

- Aph.Rep2.S5
Total number of reads: 46 565 770
Uniquely mapped reads (hg19): 26 477 903
Length of reads: 2x43

- Aph.Rep2.S6G2M
Total number of reads: 43 858 756
Uniquely mapped reads (hg19): 26 250 957
Length of reads: 2x43

| | |
|---|---|
| | |
| Antibodies | anti-BrdU monoclonal antibody (BD Biosciences, 347580) |
| Peak calling parameters | We did not perform the peak calling in our study since we aim to compare the genome-wide replication dynamics revealed by Repli-Seq between cells grown with or without aphidicolin treatment. |
| Data quality | The back-ground level and signal/noise ratio were calculated for each sample as previous described (CHEN Genome Res 2010). |
| Software | The data were analyzed using custom script written in Python (v2.7.9) and R (v3.4.4). The computer codes are available on the GitHub repositories of the team (https://github.com/CL-CHEN-Lab/). |

## Flow Cytometry

### Plots

Confirm that:

- [ ] The axis labels state the marker and fluorochrome used (e.g. CD4-FITC).
- [ ] The axis scales are clearly visible. Include numbers along axes only for bottom left plot of group (a 'group' is an analysis of identical markers).
- [ ] All plots are contour plots with outliers or pseudocolor plots.
- [ ] A numerical value for number of cells or percentage (with statistics) is provided.

### Methodology

| | |
|---|---|
| Sample preparation | The flow cytometry was used to selected the cells at different periods of S phase for the Repli-Seq experiments. And it was not used to generate plots showing new results in the manuscript. The above flow cytometry checklist is therefore not applied to our study.<br>Cells were fixed in 70% ethanol and incubated overnight at 4°C in the presence of 15 ug/ml Hoescht 33342 (ThermoFisher Scientific, H3570). Cells were re-suspended in 1X PBS and sorted in six fractions at a time by flow cytometry based on their DNA content using a BD Biosciences INFLUX cell sorter. To check the fractionation quality, the post-sorted cells, already stained with Hoescht 33342, were directly re-analyzed by flow cytometry. |
| Instrument | BD Biosciences INFLUX cell sorter |
| Software | BD FACS software 1.2.0.142 |
| Cell population abundance | - For untreated cells, G1/S1 30%, S2 6.4%, S3 5.8%, S4 4.8%, S5 5.3%, S6/G2/M 10.1% - For cells treated with Aph, G1/S1 32.3%, S2 11.4%, S3 7%, S4 4.8%, S5 4.6%, S6/G2/M 11.6%. A minimum of 2.106 cells was sorted for each fraction. |
| Gating strategy | The 1rst gating was done on the morphology (FSC/SSC). A 2nd gating was done to select single cell (Pulse Width/DNA content). Then, cells were sorted in 6 fractions G1/S1, S2, S3, S4, S5, S6/G2/M according to their DNA content. |

- [x] Tick this box to confirm that a figure exemplifying the gating strategy is provided in the Supplementary Information.
