# Supplementary material for: Transcription-mediated organization of the replication initiation program across large genes sets common fragile sites genome-wide
Source: Nat Commun. 2019 Dec 13;10:5693. doi: 10.1038/s41467-019-13674-5 (PMC6911102; doi:10.1038/s41467-019-13674-5)
Supplement: Supplementary file 8 — Source Data [file 41467_2019_13674_MOESM8_ESM.zip › Sources_Data.NCOMMS-19-27604A/Sources_Data.SupplementaryFigure1.zip!/SupplementaryFigure1/S1d/D_Common_SDR_between_normalization_methods.html]

Amount of common SDR between normalizations


# Amount of common SDR between normalizations

#### Sami EL HILALI

#### 26 novembre 2018

- 0 - IMPORTS :
- 1 - DATA INPUT :
- 2 - DATA INTEGRATION :
  - 2.1 - Calculate SDR for 15M :
  - 2.2 - Calculate SDR for mtDNA :
  - 2.3 - Calculate SDR for background\_level normalization :
  - 2.4 - Calculate SDR intersections :
- 3 - PLOT :

# 0 - IMPORTS :

```
library(dplyr)
```

This analysis features bedtools v2.25.0

# 1 - DATA INPUT :

```
### NT
NT.15M <- Rscripts.publi.714717::load_repliseq_df("../../DATA/REPLISEQ/NT1/50kb_adj_15M","NT1")
NT.mDNA <- Rscripts.publi.714717::load_repliseq_df("../../DATA/REPLISEQ/NT1/50kb_adj_mDNA","NT1")
NT.background <- Rscripts.publi.714717::load_repliseq_df("../../DATA/REPLISEQ/NT1/50kb_adj_noise_norm","NT1")

### Aph
Aph.15M <- Rscripts.publi.714717::load_repliseq_df("../../DATA/REPLISEQ/Aph1/50kb_adj_15M","Aph1")
Aph.mDNA <- Rscripts.publi.714717::load_repliseq_df("../../DATA/REPLISEQ/Aph1/50kb_adj_mDNA","Aph1")
Aph.background <- Rscripts.publi.714717::load_repliseq_df("../../DATA/REPLISEQ/Aph1/50kb_adj_noise_norm","Aph1")
# head(NT.background)
# head(Aph.background)
```

# 2 - DATA INTEGRATION :

### 2.1 - Calculate SDR for 15M :

```
ecdf_fun <- function(x,perc) ecdf(x)(perc)

calculate_SDR <- function(Aph,NT,condition) {
  
  ##### -2 ; 20 & 40 in 15M correspond to the following quantiles :
  Diff.NT.Aph <- Rscripts.publi.714717::compare_repliseq(Aph, NT)
  # Diff.NT.Aph$URI <- round(Diff.NT.Aph$URI, digits = 3)
  Diff.NT.Aph$URI[Diff.NT.Aph$MeanXY <= 0.5] <- NA
  
  # write.table(Diff.NT.Aph,file = paste0("OUTPUTS/URI.",condition,".tab"),quote= FALSE, row.names = FALSE, col.names = FALSE )
  
  threshold.mean.up <- round(as.numeric(unname(quantile(Diff.NT.Aph$MeanXY,0.9460595,na.rm = TRUE))),digits = 2)
  threshold.mean.down <- round(as.numeric(unname(quantile(Diff.NT.Aph$MeanXY,0.1022647,na.rm = TRUE))),digits = 2)
  threshold.URI <- round(as.numeric(unname(quantile(Diff.NT.Aph$URI,0.006611774,na.rm = TRUE))),digits = 2)
  

  Delayed_windows <- subset(Diff.NT.Aph,MeanXY > threshold.mean.down & MeanXY < threshold.mean.up & URI <= threshold.URI) %>%
    select(chr,start,stop,URI)
  ### Write Delayed windows bed :
  file_name = paste0("OUTPUTS/SDW.",condition,".bed")
  write.table(x = Delayed_windows,file = file_name,quote = FALSE, sep = "\t",row.names = FALSE, col.names = FALSE)
  
  ### we take relative ratios for further analysis, the first step of calculation is : 
  print(ecdf_fun(Diff.NT.Aph$URI,-2))
  print(ecdf_fun(Diff.NT.Aph$MeanXY,20))
  print(ecdf_fun(Diff.NT.Aph$MeanXY,40))
  
}

calculate_SDR(Aph.15M,NT.15M,"15M")
```

```
## [1] 0.006611774
## [1] 0.1022647
## [1] 0.9460595
```

```
### 1 - merge adjacent SDW into SDR
bedtools merge -i OUTPUTS/SDW.15M.bed | awk '{if (($3 - $2) > 50000) { print $1"\t"$2"\t"$3}}' | bedtools merge -i - -d 250000 | bedtools sort -i - > OUTPUTS/temp.SDR.15M.bed

### 2 - write SDW with removing those clustering in SDR
bedtools merge -i OUTPUTS/SDW.15M.bed | awk '( ($3 - $2) == 50000 ) { print $1"\t"$2"\t"$3}' | bedtools sort -i - > OUTPUTS/temp.SDW.minus.SDR.15M.bed

### 3 - merge SDW at max 250kb distance to SDR with SDR
bedtools closest -a OUTPUTS/temp.SDR.15M.bed -D "b" -b OUTPUTS/temp.SDW.minus.SDR.15M.bed | awk '{ if(($7 <= 250000) && ($7 >= -250000) && ($4 != ".")) {print}}' | awk '{if ($7 > 0) {print $1"\t"$2"\t"$6} else {print $1"\t"$5"\t"$3}}' | bedtools sort -i - > OUTPUTS/temp.SDR.plus.closeSDW.15M.bed

cat OUTPUTS/temp.SDR.15M.bed OUTPUTS/temp.SDR.plus.closeSDW.15M.bed | bedtools sort -i - | bedtools merge -i - > OUTPUTS/SDR.15M.bed

### remove temporary files :
rm OUTPUTS/temp.*
rm OUTPUTS/SDW.15M.bed
```

### 2.2 - Calculate SDR for mtDNA :

```
calculate_SDR <- function(Aph,NT,condition) {
  
  Diff.NT.Aph <- Rscripts.publi.714717::compare_repliseq(Aph, NT)

  Diff.NT.Aph$URI[Diff.NT.Aph$MeanXY <= 0.5] <- NA
  
  # write.table(Diff.NT.Aph,file = paste0("OUTPUTS/URI.",condition,".tab"),quote= FALSE, row.names = FALSE, col.names = FALSE )
  
  ##### -2 ; 20 & 40 in 15M correspond to the following quantiles :
  threshold.mean.up <- round(as.numeric(unname(quantile(Diff.NT.Aph$MeanXY,0.9460595,na.rm = TRUE))),digits = 2)
  threshold.mean.down <- round(as.numeric(unname(quantile(Diff.NT.Aph$MeanXY,0.1022647,na.rm = TRUE))),digits = 2)
  threshold.URI <- round(as.numeric(unname(quantile(Diff.NT.Aph$URI,0.006611774,na.rm = TRUE))),digits = 2)
  
  Delayed_windows <- subset(Diff.NT.Aph,MeanXY > threshold.mean.down & MeanXY < threshold.mean.up & URI <= threshold.URI) %>%
    select(chr,start,stop,URI)
  ### Write Delayed windows bed :
  file_name = paste0("OUTPUTS/SDW.",condition,".bed")
  write.table(x = Delayed_windows,file = file_name,quote = FALSE, sep = "\t",row.names = FALSE, col.names = FALSE)
  
  print(paste0("Thresholds : ",threshold.mean.down,", ",threshold.mean.up,", ",threshold.URI))
}

calculate_SDR(Aph.mDNA,NT.mDNA,"mDNA")
```

```
## [1] "Thresholds : 1296.02, 2596.15, -1.99"
```

```
# calculate_SDR(Aph.background,NT.background,"background")
```

```
### 1 - merge adjacent SDW into SDR
bedtools merge -i OUTPUTS/SDW.mDNA.bed | awk '{if (($3 - $2) > 50000) { print $1"\t"$2"\t"$3}}' | bedtools merge -i - -d 250000 | bedtools sort -i - > OUTPUTS/temp.SDR.mDNA.bed

### 2 - write SDW with removing those clustering in SDR
bedtools merge -i OUTPUTS/SDW.mDNA.bed | awk '( ($3 - $2) == 50000 ) { print $1"\t"$2"\t"$3}' | bedtools sort -i - > OUTPUTS/temp.SDW.minus.SDR.mDNA.bed

### 3 - merge SDW at max 250kb distance to SDR with SDR
bedtools closest -a OUTPUTS/temp.SDR.mDNA.bed -D "b" -b OUTPUTS/temp.SDW.minus.SDR.mDNA.bed | awk '{ if(($7 <= 250000) && ($7 >= -250000) && ($4 != ".")) {print}}' | awk '{if ($7 > 0) {print $1"\t"$2"\t"$6} else {print $1"\t"$5"\t"$3}}' | bedtools sort -i - > OUTPUTS/temp.SDR.plus.closeSDW.mDNA.bed

cat OUTPUTS/temp.SDR.mDNA.bed OUTPUTS/temp.SDR.plus.closeSDW.mDNA.bed | bedtools sort -i - | bedtools merge -i - > OUTPUTS/SDR.mDNA.bed

### remove temporary files :
rm OUTPUTS/temp.*
rm OUTPUTS/SDW.mDNA.bed
```

### 2.3 - Calculate SDR for background\_level normalization :

```
calculate_SDR <- function(Aph,NT,condition) {
  
  Diff.NT.Aph <- Rscripts.publi.714717::compare_repliseq(Aph, NT)

  Diff.NT.Aph$URI[Diff.NT.Aph$MeanXY <= 0.5] <- NA
  
  # write.table(Diff.NT.Aph,file = paste0("OUTPUTS/URI.",condition,".tab"),quote= FALSE, row.names = FALSE, col.names = FALSE )
  
  ##### -2 ; 20 & 40 in 15M correspond to the following quantiles :
  threshold.mean.up <- round(as.numeric(unname(quantile(Diff.NT.Aph$MeanXY,0.9460595,na.rm = TRUE))),digits = 2)
  threshold.mean.down <- round(as.numeric(unname(quantile(Diff.NT.Aph$MeanXY,0.1022647,na.rm = TRUE))),digits = 2)
  threshold.URI <- round(as.numeric(unname(quantile(Diff.NT.Aph$URI,0.006611774,na.rm = TRUE))),digits = 2)
  
  Delayed_windows <- subset(Diff.NT.Aph,MeanXY > threshold.mean.down & MeanXY < threshold.mean.up & URI <= threshold.URI) %>%
    select(chr,start,stop,URI)
  ### Write Delayed windows bed :
  file_name = paste0("OUTPUTS/SDW.",condition,".bed")
  write.table(x = Delayed_windows,file = file_name,quote = FALSE, sep = "\t",row.names = FALSE, col.names = FALSE)
  
  print(paste0("Thresholds : ",threshold.mean.down,", ",threshold.mean.up,", ",threshold.URI))
}

calculate_SDR(Aph.background,NT.background,"back_level")
```

```
## [1] "Thresholds : 3080.22, 6218.93, -1.89"
```

```
# calculate_SDR(Aph.background,NT.background,"background")
```

```
### 1 - merge adjacent SDW into SDR
bedtools merge -i OUTPUTS/SDW.back_level.bed | awk '{if (($3 - $2) > 50000) { print $1"\t"$2"\t"$3}}' | bedtools merge -i - -d 250000 | bedtools sort -i - > OUTPUTS/temp.SDR.back_level.bed

### 2 - write SDW with removing those clustering in SDR
bedtools merge -i OUTPUTS/SDW.back_level.bed | awk '( ($3 - $2) == 50000 ) { print $1"\t"$2"\t"$3}' | bedtools sort -i - > OUTPUTS/temp.SDW.minus.SDR.back_level.bed

### 3 - merge SDW at max 250kb distance to SDR with SDR
bedtools closest -a OUTPUTS/temp.SDR.back_level.bed -D "b" -b OUTPUTS/temp.SDW.minus.SDR.back_level.bed | awk '{ if(($7 <= 250000) && ($7 >= -250000) && ($4 != ".")) {print}}' | awk '{if ($7 > 0) {print $1"\t"$2"\t"$6} else {print $1"\t"$5"\t"$3}}' | bedtools sort -i - > OUTPUTS/temp.SDR.plus.closeSDW.back_level.bed

cat OUTPUTS/temp.SDR.back_level.bed OUTPUTS/temp.SDR.plus.closeSDW.back_level.bed | bedtools sort -i - | bedtools merge -i - > OUTPUTS/SDR.back_level.bed

### remove temporary files :
rm OUTPUTS/temp.*
rm OUTPUTS/SDW.back_level.bed
```

### 2.4 - Calculate SDR intersections :

```
### Background & 15Mreads & mtDNA : 44

bedtools intersect -a OUTPUTS/SDR.back_level.bed -b OUTPUTS/SDR.15M.bed | bedtools intersect -a - -b OUTPUTS/SDR.mDNA.bed | wc -l

### Background & 15Mreads : 9

bedtools intersect -a OUTPUTS/SDR.back_level.bed -b OUTPUTS/SDR.15M.bed | bedtools intersect -a - -b OUTPUTS/SDR.mDNA.bed -wao | awk '($4 == ".") { print $1"\t"$2"\t"$3}' | wc -l


### Background & mtDNA : 0

bedtools intersect -a OUTPUTS/SDR.back_level.bed -b OUTPUTS/SDR.mDNA.bed | bedtools intersect -a - -b OUTPUTS/SDR.15M.bed -wao | awk '($4 == ".") { print $1"\t"$2"\t"$3}' | wc -l


### 15Mreads & mtDNA : 0

bedtools intersect -a OUTPUTS/SDR.15M.bed -b OUTPUTS/SDR.mDNA.bed | bedtools intersect -a - -b OUTPUTS/SDR.back_level.bed -wao | awk '($4 == ".") { print $1"\t"$2"\t"$3}' | wc -l

### Background : 2

bedtools intersect -a OUTPUTS/SDR.back_level.bed -b OUTPUTS/SDR.15M.bed -wao | awk '($4 == ".") { print $1"\t"$2"\t"$3}' | bedtools intersect -a - -b OUTPUTS/SDR.mDNA.bed -wao | awk '($4 == ".") { print $1"\t"$2"\t"$3}' | wc -l

### 15Mreads : 4

bedtools intersect -a OUTPUTS/SDR.15M.bed -b OUTPUTS/SDR.back_level.bed -wao | awk '($4 == ".") { print $1"\t"$2"\t"$3}' | bedtools intersect -a - -b OUTPUTS/SDR.mDNA.bed -wao | awk '($4 == ".") { print $1"\t"$2"\t"$3}' | wc -l

### mtDNA :  1

bedtools intersect -a OUTPUTS/SDR.mDNA.bed -b OUTPUTS/SDR.back_level.bed -wao | awk '($4 == ".") { print $1"\t"$2"\t"$3}' | bedtools intersect -a - -b OUTPUTS/SDR.15M.bed -wao | awk '($4 == ".") { print $1"\t"$2"\t"$3}' | wc -l
```

```
## 44
## 9
## 0
## 0
## 2
## 4
## 1
```

# 3 - PLOT :
