# Supplementary material for: Transcription-mediated organization of the replication initiation program across large genes sets common fragile sites genome-wide
Source: Nat Commun. 2019 Dec 13;10:5693. doi: 10.1038/s41467-019-13674-5 (PMC6911102; doi:10.1038/s41467-019-13674-5)
Supplement: Supplementary file 8 — Source Data [file 41467_2019_13674_MOESM8_ESM.zip › Sources_Data.NCOMMS-19-27604A/Sources_Data.SupplementaryFigure1.zip!/SupplementaryFigure1/S1c/C_Clustering_SDW.html]

Clustering of SDW


# Clustering of SDW

#### Sami EL HILALI

#### 25 octobre 2018

- 0 - IMPORTS :
- 1 - DATA INPUT :
- 2 - DATA INTEGRATION :
  - 2.1 - Calculate distances to closest SDW :
  - 2.2 - Random simultation genome wide :
- 3 - PLOT :

# 0 - IMPORTS :

```
library(dplyr)
library(ggplot2)
library(ggthemes)
```

# 1 - DATA INPUT :

this analysis features significantly delayed windows (Delayed\_windows.50kb.bed) and S50 for the random simulation

```
S50 <- read.csv("../../DATA/S50/NT1.S50.bed", sep = "", header = FALSE) %>%
  rename(chr = V1, start = V2, stop = V3, S50 = V4)
```

# 2 - DATA INTEGRATION :

### 2.1 - Calculate distances to closest SDW :

```
euc.dist <- function(x1, x2) sqrt(sum((x1 - x2) ^ 2))

### Calculate distances to closest SDW : 

system(command = "bedtools closest -a ../../DATA/SDRW/Delayed_windows.50kb.bed -b ../../DATA/SDRW/Delayed_windows.50kb.bed -io > OUTPUTS/Delayed_windows.closest.bed")

SDW.closest <- read.csv("OUTPUTS/Delayed_windows.closest.bed", sep ="", header = FALSE) %>%
  rename(chr = V1, start.x = V2, stop.x = V3, start.y = V6, stop.y = V7) %>%
  select(chr,start.x,stop.x,start.y,stop.y) %>%
  mutate(mid.x = (start.x + stop.x) /2) %>%
  mutate(mid.y = (start.y + stop.y) /2) %>%
  rowwise %>%
  mutate(dist = euc.dist(mid.x,mid.y))

SDW.closest <- SDW.closest %>%
  group_by(chr,start.x,stop.x) %>%
  summarise(Dist = min(dist))
```

### 2.2 - Random simultation genome wide :

```
### Control = Random simulation genome-wide :

control.closest <- NULL
# set.seed(1031) 
for ( i in c(1:1000)) {
  temp <- sample_n(S50,331)
  write.table(temp,"OUTPUTS/Random.windows.bed",sep="\t",row.names = FALSE,col.names = FALSE, quote = FALSE)
  system(command = "bedtools sort -i OUTPUTS/Random.windows.bed -faidx ../../DATA/Hg19/chr_names.txt > OUTPUTS/Random.windows.sorted.bed ")
  system(" bedtools closest -a OUTPUTS/Random.windows.sorted.bed -b OUTPUTS/Random.windows.sorted.bed -io > OUTPUTS/Random.windows.closest.bed")
  control.temp <- read.csv("OUTPUTS/Random.windows.closest.bed", sep ="", header = FALSE) %>%
    rename(chr = V1, start.x = V2, stop.x = V3, start.y = V6, stop.y = V7) %>%
    select(chr,start.x,stop.x,start.y,stop.y) %>%
    mutate(mid.x = (start.x + stop.x) /2) %>%
    mutate(mid.y = (start.y + stop.y) /2) %>%
    rowwise %>%
    mutate(dist = euc.dist(mid.x,mid.y)) 

control.temp <- control.temp %>%
  group_by(chr,start.x,stop.x) %>%
  summarise(Dist = min(dist))

temp <- control.temp
control.closest <- rbind(control.closest,control.temp)
}

###
system(command = "rm OUTPUTS/Random.windows.bed OUTPUTS/Random.windows.sorted.bed")

### Append SDW with Control :

SDW.closest <- SDW.closest %>%
  mutate(group = "SDW")

control.closest <- control.closest %>%
  mutate(group = "Control")

control_and_SDW <- rbind(SDW.closest,control.closest)


### Export data table :
write.table(control_and_SDW, file = "OUTPUTS/control_and_sdw.txt",sep="\t",quote=FALSE,row.names = FALSE)
###
```

# 3 - PLOT :

```
############## PLOT #############

p <- ggplot(control_and_SDW,aes(x="",y= log10(Dist / 1000), color = group)) +
  geom_boxplot(size = 1.5) +
  theme_bw() +
  scale_color_manual(values = c("black","darkolivegreen4")) +
  geom_hline(yintercept = log10(250), linetype = "dashed", size = 2) +
  theme(axis.title.y = element_text(size = 22, face = "bold.italic"),
        axis.text = element_text(size = 18, color = "black"),
        legend.text = element_text(size = 18),
        panel.border = element_rect(fill = NA, size = 3,color = "black")) +
  labs(x="", y = "log10(Distance (kb))",color="")

### Export plot as PNG :

ggsave(file = "OUTPUTS/S1C_left.png", plot = p)

print(p)
```
