# Supplementary material for: Transcription-mediated organization of the replication initiation program across large genes sets common fragile sites genome-wide
Source: Nat Commun. 2019 Dec 13;10:5693. doi: 10.1038/s41467-019-13674-5 (PMC6911102; doi:10.1038/s41467-019-13674-5)
Supplement: Supplementary file 8 — Source Data [file 41467_2019_13674_MOESM8_ESM.zip › Sources_Data.NCOMMS-19-27604A/Sources_Data.SupplementaryFigure1.zip!/SupplementaryFigure1/S1c/C_Amount_SDW_per_SDR.html]

Amount of SDW per SDR


# Amount of SDW per SDR

#### Sami EL HILALI

#### 22 novembre 2018

- 0 - IMPORTS :
- 1 - DATA INPUT :
- 2 - DATA INTEGRATION :
- 3 - PLOT :

# 0 - IMPORTS :

```
library(dplyr)
library(ggplot2)
library(ggthemes)
```

# 1 - DATA INPUT :

```
SDR <- read.csv("../../DATA/SDRW/SDR.bed", header = FALSE, sep ="") %>%
  rename(chr = V1, start = V2, stop =V3)
```

# 2 - DATA INTEGRATION :

```
SDR <- SDR  %>%
  mutate(size_kb = (stop - start) / 1000) %>%
  mutate(Amount_SDW = size_kb / 50) %>%
  group_by(Amount_SDW) %>%
  summarise(n = n())
```

# 3 - PLOT :

```
############## PLOT #############

p <- ggplot(SDR,aes(x= Amount_SDW, y = n)) +
  geom_col() +
  theme_bw() +
  coord_cartesian(xlim = c(0,18)) +
  theme(axis.text = element_text(size = 18,color = "black"),
        axis.title = element_text(size = 22, color = "black"),
        panel.border = element_rect(fill = NA, size = 3,color = "black")) +
  labs(x = "Number of SDW per SDR", y = "Number of SDRs")


### Export plot as PNG :

ggsave(file = "OUTPUTS/S1C_right.png", plot = p)

print(p)
```
