# Supplementary material for: Transcription-mediated organization of the replication initiation program across large genes sets common fragile sites genome-wide
Source: Nat Commun. 2019 Dec 13;10:5693. doi: 10.1038/s41467-019-13674-5 (PMC6911102; doi:10.1038/s41467-019-13674-5)
Supplement: Supplementary file 8 — Source Data [file 41467_2019_13674_MOESM8_ESM.zip › Sources_Data.NCOMMS-19-27604A/Sources_Data.SupplementaryFigure1.zip!/SupplementaryFigure1/S1b/B_Timing_Jeff_Vs_GM.html]

Timing S50 Jeff Vs GM


# Timing S50 Jeff Vs GM

#### Sami EL HILALI

#### 8 mai 2018

# 0 - IMPORTS :

```
### IMPORTS :
library(dplyr)
library(ggplot2)
library(ggthemes)
library(reshape2)
library(corrplot)
library(RColorBrewer)
library(Repliseq)
```

# 1 - DATA INPUT :

Jeff2 and Aph S50 was calculated based on 50kb adj profiles normalized to 15M reads.

# 2 - DATA INTEGRATION :

# 3 - PLOT :

```
############## PLOT #############
### Calculation of the p-values associated with a correlation matrix
cor.mtest <- function(mat, ...) {
    mat <- as.matrix(mat)
    n <- ncol(mat)
    p.mat<- matrix(NA, n, n)
    diag(p.mat) <- 0
    for (i in 1:(n - 1)) {
        for (j in (i + 1):n) {
            tmp <- cor.test(mat[, i], mat[, j], ...)
            p.mat[i, j] <- p.mat[j, i] <- tmp$p.value
        }
    }
  colnames(p.mat) <- rownames(p.mat) <- colnames(mat)
  p.mat
}

replicates_correlation <- function(replicates,method = "spearman",viz = "mixed") {
  # ressource : http://www.sthda.com/french/wiki/visualiser-une-matrice-de-correlation-par-un-correlogramme
  # Calculate correlation matrix
  temp.cor <- cor(replicates,method = method)
  # Calculate p-values associated with each correlation
  p.mat <- cor.mtest(temp.cor)
  # plot the correlation matrix
  # corrplot(temp.cor,is.corr = FALSE, method="pie",type="upper",order="hclust", tl.col="black", tl.srt=45,addrect = 2,col = terrain.colors(100))
  col4 <- c("#440154FF" ,"#414487FF" ,"#2A788EFF", "#22A884FF" ,"#7AD151FF" ,"#FDE725FF")
  corrplot.mixed(temp.cor,is.corr = FALSE, tl.col="black",addrect = 2,upper.col = brewer.pal(n = 10, name = "PuOr"),lower.col=brewer.pal(n = 10, name = "PuOr"),tl.cex=0.8,number.cex = 1.2,cl.cex = 1.1)
}

png(filename="OUTPUTS/S1B.png")
replicates_correlation(replicates)
dev.off()
```

```
## png 
##   2
```

```
replicates_correlation(replicates)
```
