# Supplementary material for: Transcription-mediated organization of the replication initiation program across large genes sets common fragile sites genome-wide
Source: Nat Commun. 2019 Dec 13;10:5693. doi: 10.1038/s41467-019-13674-5 (PMC6911102; doi:10.1038/s41467-019-13674-5)
Supplement: Supplementary file 8 — Source Data [file 41467_2019_13674_MOESM8_ESM.zip › Sources_Data.NCOMMS-19-27604A/Sources_Data.SupplementaryFigure3.zip!/SupplementaryFigure3/S3a/A_Rbands_histogram.html]

R\_banding


# R\_banding

#### Sami EL HILALI

#### 27 juin 2018

- 0 - IMPORTS :
- 1 - DATA INPUT :
- 2 - DATA INTEGRATION :
- 3 - PLOT :

# 0 - IMPORTS :

```
library(dplyr)
library(ggplot2)
library(ggthemes)
```

# 1 - DATA INPUT :

```
rbands <- read.csv("R-banding.tab",sep="")
```

# 2 - DATA INTEGRATION :

NA

# 3 - PLOT :
