# Supplementary material for: Transcription-mediated organization of the replication initiation program across large genes sets common fragile sites genome-wide
Source: Nat Commun. 2019 Dec 13;10:5693. doi: 10.1038/s41467-019-13674-5 (PMC6911102; doi:10.1038/s41467-019-13674-5)
Supplement: Supplementary file 8 — Source Data [file 41467_2019_13674_MOESM8_ESM.zip › Sources_Data.NCOMMS-19-27604A/Sources_Data.SupplementaryFigure2.zip!/SupplementaryFigure2/S2b/B_Downslopes_near_SDW.html]

Down slope near SDW in long genes


# Down slope near SDW in long genes

#### Sami EL HILALI

#### 26 Mars 2019

- 0 - IMPORTS :
- 1 - DATA INPUTS :
- 2 - DATA INTEGRATION :
- 3 - PLOT :
  - 3.1 - Upstream & Downstream sizes :
  - 3.2 - Slopes :

# 0 - IMPORTS :

```
library(dplyr)
library(ggplot2)
```

# 1 - DATA INPUTS :

The script calculating the RAI over the downslopes take as input :  
- Selected\_SDW.genes.bed  
- RAI.NT1vsAph1.50kb.bed  
- sdw.full\_down\_slope.bed

```
### LOAD Down slopes sizes data :
down_slopes <- read.csv("../../DATA/Down_slopes/SDW.down_slopes.exact_gene_pos.strand.tab",header= FALSE, sep = "") %>%
  rename(GeneName = V1,upstream = V2, downstream = V3, gene_direction = V4, gene_size = V5)

down_slopes <- subset(down_slopes, upstream > 0 & downstream > 0)
```

# 2 - DATA INTEGRATION :

```
echo "gene_name,gene_orientation,slope_type,URI,mid_window,mid_SDR,start_SDR,stop_SDR" > OUTPUTS/Slope_SDW_gene.tab

awk '{if ($7 == "+") {print $1"\t"$2-($4*1000)"\t"$2+50000"\t"$6"\t"$7"\tupstream\t"$2"\t"$3"\n"$1"\t"$3-50000"\t"$3+($5*1000)"\t"$6"\t"$7"\tdownstream\t"$2"\t"$3} else {print $1"\t"$2-($5*1000)"\t"$2+50000"\t"$6"\t"$7"\tdownstream\t"$2"\t"$3"\n"$1"\t"$3-50000"\t"$3+($4*1000)"\t"$6"\t"$7"\tupstream\t"$2"\t"$3}}' ../../DATA/Down_slopes/SDW.down_slopes.bed | bedtools intersect -a - -b ../../DATA/Down_slopes/Selected_SDW.genes.bed | bedtools intersect -a - -b ../../DATA/URI/URI.NT1vsAph1.50kb.bed -wao | awk '{print $4","$5","$6","$12","($10+$11)/2","($7+$8)/2","$7","$8}' >> OUTPUTS/Slope_SDW_gene.tab
```

# 3 - PLOT :

There are 28 selected SDW which are hosted within long transcribed genes (see Table S2).

### 3.1 - Upstream & Downstream sizes :

```
############## PLOT #############

p <- ggplot(down_slopes,aes(x= upstream, y = downstream,color = gene_direction)) +
  geom_jitter(aes(size = gene_size)) +
  theme_bw() +
  geom_abline(slope = 1, intercept = 0, linetype = "dashed") +
  coord_cartesian(xlim = c(0,300),ylim = c(0,300)) +
  scale_color_manual(values = c("blue2", "deepskyblue")) +
  theme(axis.title = element_text(size = 22),
        legend.position = "top",
        axis.text = element_text(size = 18,color = "black"),
        legend.text = element_text(size = 18, color = "black"),
        legend.title = element_text(size = 18, color = "black"),
        plot.title = element_text(size = 15,face = "bold" ),
        panel.border = element_rect(fill = NA, size = 3,color = "black")) +
labs(x= "Upstream size (kb)", y = "Downstream size (kb)",color = "Gene orientation")

### Export plot as PNG :

ggsave(file = "OUTPUTS/S2B_top.png", plot = p)

print(p)
```

### 3.2 - Slopes :

```
### LOAD Down slopes  RAI data :
slope.gene <- read.csv("OUTPUTS/Slope_SDW_gene.tab") %>%
  group_by(gene_name,slope_type) %>%
  mutate(mid_window_scaled = (mid_window - mid_SDR)/1000) %>%
  mutate(start_window_scaled = (mid_window - start_SDR)/1000) %>%
  mutate(start_window_scaled = replace(start_window_scaled,gene_orientation == "+" && slope_type == "downstream",(mid_window - stop_SDR + 25000)/1000)) %>%
  mutate(start_window_scaled = replace(start_window_scaled,gene_orientation == "+" && slope_type == "upstream",(mid_window - start_SDR - 25000)/1000)) %>%
  mutate(start_window_scaled = replace(start_window_scaled,gene_orientation == "-" && slope_type == "downstream",(mid_window - start_SDR - 25000)/1000)) %>%
  mutate(start_window_scaled = replace(start_window_scaled,gene_orientation == "-" && slope_type == "upstream",(mid_window - stop_SDR + 25000)/1000))

############## PLOT #############
p <- ggplot(slope.gene,aes(x= start_window_scaled,y = URI)) +
  geom_line(aes(color = gene_name,linetype = slope_type)) +
  coord_cartesian(ylim = c(-3,2.5)) +
  theme_bw() +
  geom_vline(xintercept = 0,linetype = "dotted") +
  geom_hline(yintercept = -2, linetype = "dashed") +
  theme(axis.title = element_text(size = 22),
        legend.position = "none",
        axis.text = element_text(size = 18,color = "black"),
        legend.text = element_text(size = 18, color = "black"),
        legend.title = element_text(size = 18, color = "black"),
        plot.title = element_text(size = 15,face = "bold" ),
        panel.border = element_rect(fill = NA, size = 3,color = "black")) +
  labs(x="Position relative to T-SDW (kb)")


### Export plot as PNG :

ggsave(file = "OUTPUTS/S2B_bottom.png", plot = p)

print(p)
```
