# Supplementary material for: Transcription-mediated organization of the replication initiation program across large genes sets common fragile sites genome-wide
Source: Nat Commun. 2019 Dec 13;10:5693. doi: 10.1038/s41467-019-13674-5 (PMC6911102; doi:10.1038/s41467-019-13674-5)
Supplement: Supplementary file 8 — Source Data [file 41467_2019_13674_MOESM8_ESM.zip › Sources_Data.NCOMMS-19-27604A/Sources_Data.Figure1.zip!/Figure1/1b/B_Delay_Vs_Timing.html]

Figure 1B


# Figure 1B

#### Sami EL HILALI

#### 6 novembre 2018

- 0 - IMPORTS :
- 1 - DATA INPUT :
- 2 - DATA INTEGRATION :
- 3 - PLOT

For this report, the sum of the samples were normalized to 15M reads.

# 0 - IMPORTS :

```
library(dplyr)
library(ggplot2)
```

# 1 - DATA INPUT :

```
### Data paths :

NT1_path <- "../../DATA/REPLISEQ/NT1/50kb_adj_15M"
Aph1_path <- "../../DATA/REPLISEQ/Aph1/50kb_adj_15M"
S50_path <- "../../DATA/S50/NT1.S50.bed"

### Load data :
NT <- Rscripts.publi.714717::load_repliseq_df(NT1_path,"NT1")

Aph <- Rscripts.publi.714717::load_repliseq_df(Aph1_path,"Aph1")

S50 <- read.csv(S50_path,header=FALSE,sep="") %>%
  rename(chr = V1, start = V2, stop = V3, S50 = V4)
```

# 2 - DATA INTEGRATION :

```
### Compare the 2 repliseq assays --> Calculates RAI
NT.vs.Aph <- Rscripts.publi.714717::compare_repliseq(Aph,NT)

### merge S50 and Delay index
temp <- merge(S50,NT.vs.Aph) %>%
    mutate(Timing = "Early") %>%
    mutate(Timing = replace(Timing,S50 >= 0.25, "Mid-Early")) %>%
    mutate(Timing = replace(Timing,S50 >= 0.5, "Mid-Late")) %>%
    mutate(Timing = replace(Timing,S50 >= 0.75, "Late"))

temp$Timing <- factor(temp$Timing,levels=c("Early","Mid-Early","Mid-Late","Late"))

### Process data frame (to put in a script where we generate the data)
temp <- temp %>%
  mutate(delay="Significant") %>%
  mutate(delay = replace(delay,URI > -2,"Yes")) %>%
  mutate(delay = replace(delay,URI >= 0,"No")) %>%
  mutate(delay = replace(delay,MeanXY < 20, "NA")) %>%
  mutate(delay = replace(delay,MeanXY > 40, "NA"))

### Export data to csv
write.table(temp, file = "OUTPUTS/Delay_Vs_Timing.txt",sep="\t",quote=FALSE,row.names = FALSE)
###
```

# 3 - PLOT

```
### Filter NAs and reprocess factor before drawing
temp <- subset(temp,delay != "NA")
temp$delay <- factor(temp$delay,levels =c("No","Yes","Significant"))

### some stats
temp.amount <- temp %>%
  group_by(Timing,delay) %>%
  summarise(Amount = n() * 0.05)
### Write table for the plot
write.table(temp.amount,file="OUTPUTS/Plot.table.txt",row.names = FALSE, quote = FALSE,sep = "\t")
###


############## PLOT #############

p <- ggplot(temp.amount,aes(x=Timing,Amount,fill=delay)) +
  geom_col(color="black",size=1,width = 0.4,position="dodge") +
  theme_bw() +
  scale_fill_manual(values = c("grey","#f9cf9c","red")) +
  theme(legend.position = "top",legend.text = element_text(size = 18),legend.title = element_text(size=20),
        axis.title = element_text(size = 22),
        axis.text.x = element_text(size=18,color="black"),
        axis.text.y = element_text(size = 18,color = "black"),
        strip.text = element_blank(),
        panel.border = element_rect(fill = NA, size = 3,color = "black")) +
  labs(fill = "Delay : ", x ="", y = "Sum of 50 kb windows (Mb)")

### Export plot as PNG :

ggsave(file = "OUTPUTS/1B.png", plot = p)

print(p)
```
