# Supplementary material for: Transcription-mediated organization of the replication initiation program across large genes sets common fragile sites genome-wide
Source: Nat Commun. 2019 Dec 13;10:5693. doi: 10.1038/s41467-019-13674-5 (PMC6911102; doi:10.1038/s41467-019-13674-5)
Supplement: Supplementary file 8 — Source Data [file 41467_2019_13674_MOESM8_ESM.zip › Sources_Data.NCOMMS-19-27604A/Sources_Data.Figure1.zip!/Figure1/1e/E_SDRW_vs_genes_and_transcripts.html]

SDW Vs genes & transcripts


# SDW Vs genes & transcripts

#### Sami EL HILALI

#### 25 octobre 2018

# 0 - IMPORTS :

```
library(dplyr)
library(ggplot2)
library(ggthemes)
```

# 1 - DATA INPUT :

```
SDR <- data.frame(variable =c("transcription >= 300kb, 1 large gene",
                               "transcription >= 300kb, gene(s) < 300kb",
                               "transcription >= 300kb no annotation",
                               "transcription < 300kb"),
                   value = c(31,11,4,11))

SDW <- data.frame(variable =c("transcription >= 300kb, 1 large gene",
                               "transcription >= 300kb, gene(s) < 300kb",
                               "transcription >= 300kb no annotation",
                               "transcription < 300kb"),
                   value = c(31,9,5,71))
```

# 2 - DATA INTEGRATION :

NA

# 3 - PLOT :

### 3.1 - SDR :

```
SDR$variable <- factor(SDR$variable,levels = unique(SDR$variable))
knitr::kable(SDR)
```

| variable | value |
| --- | --- |
| transcription >= 300kb, 1 large gene | 31 |
| transcription >= 300kb, gene(s) < 300kb | 11 |
| transcription >= 300kb no annotation | 4 |
| transcription < 300kb | 11 |

```
############## PLOT #############
p <- ggplot(SDR,aes(x="",y=value,fill=variable)) +
  geom_bar(width = 1, stat = "identity",color="white",size = 2) +
  coord_polar("y", start=0) +
  theme_tufte() +
  scale_fill_manual(values = c("#df5a01","#f48616","#ec7a66","grey")) +
  theme(plot.title = element_text(size =25,face="bold"),
        axis.text = element_blank(),
        axis.title = element_blank(),
        legend.text = element_text(size = 18),
        legend.title = element_blank()) +
  labs(title="")


### Export plot as PNG :

ggsave(file = "OUTPUTS/1E_left.png", plot = p)

print(p)
```

### 3.2 - SDW :

```
SDW$variable <- factor(SDW$variable,levels = unique(SDW$variable))
knitr::kable(SDW)
```

| variable | value |
| --- | --- |
| transcription >= 300kb, 1 large gene | 31 |
| transcription >= 300kb, gene(s) < 300kb | 9 |
| transcription >= 300kb no annotation | 5 |
| transcription < 300kb | 71 |

```
############## PLOT #############
p <- ggplot(SDW,aes(x="",y=value,fill=variable)) +
  geom_bar(width = 1, stat = "identity",color="white",size = 2) +
  coord_polar("y", start=0) +
  theme_tufte() +
  scale_fill_manual(values = c("#df5a01","#f48616","#ec7a66","grey")) +
  theme(plot.title = element_text(size =25,face="bold"),
        axis.text = element_blank(),
        axis.title = element_blank(),
        legend.text = element_text(size = 18),
        legend.title = element_blank()) +
  labs(title="")

### Export plot as PNG :

ggsave(file = "OUTPUTS/1E_right.png", plot = p)

print(p)
```
