# Supplementary material for: Transcription-mediated organization of the replication initiation program across large genes sets common fragile sites genome-wide
Source: Nat Commun. 2019 Dec 13;10:5693. doi: 10.1038/s41467-019-13674-5 (PMC6911102; doi:10.1038/s41467-019-13674-5)
Supplement: Supplementary file 8 — Source Data [file 41467_2019_13674_MOESM8_ESM.zip › Sources_Data.NCOMMS-19-27604A/Sources_Data.Figure1.zip!/Figure1/1d/D_Genes_Vs_SDRW_1kb.html]

Genes Vs SDR & SDW


# Genes Vs SDR & SDW

#### Sami EL HILALI

#### 14 novembre 2018

- 0 - IMPORTS :
- 1 - DATA INPUT :
- 2 - DATA INTEGRATION :
  - 2.1 - Bash integration of Genes, RNA-seq, S50, GRO-seq, and SDRW :
  - 2.2 - Reshape data :
  - 2.3 - Statistical tests :
- 3 - PLOT :

# 0 - IMPORTS :

This analysis relies on data preprocessing performed with bashscripts involving :  
\* bedtools v2.25.0  
\* bigWigToBedGraph (obtained from http://hgdownload.soe.ucsc.edu/admin/exe/)

```
library(dplyr)
library(ggplot2)
```

# 1 - DATA INPUT :

This analysis features publicaly available data :  
- gencode V7 annotation (GRCh37, version 7,Ensembl 62) - RNA-seq data (GEO : GSM758559) - GRO-seq data (GEO : GSM1480326)

# 2 - DATA INTEGRATION :

### 2.1 - Bash integration of Genes, RNA-seq, S50, GRO-seq, and SDRW :

```
### Script provided @ Fig1/1D/
# ./Integrate_DATA_FIg_1D.sh
```

### 2.2 - Reshape data :

The GRO-seq signal is calculated as 10^median(GRO-seq) with GRO-seq being the read count over 1kb along all genes.

```
### Load integrated data :

genes.alldata <- read.csv("OUTPUTS/protein_coding_genes.all_data.bed",header = FALSE, sep = "") %>%
  rename(chr = V1, start =V2, stop = V3, strand = V4, geneName = V5, RPKM1 = V6, RPKM2 = V7, S50 = V8, GROseq = V9, sdr = V10, sdw = V11) %>%
  mutate(RPKM = (RPKM1 + RPKM2) / 2)
genes.alldata$S50 <- as.numeric(as.character(genes.alldata$S50))

### Reshape data :
genes.reshaped_data <- genes.alldata %>%
  group_by(chr,strand,geneName) %>%
  summarise(start = min(start), stop = max(stop),
            RPKM1 = mean(RPKM1),  RPKM2 = mean(RPKM2), RPKM = mean(RPKM),
            S50 = mean(S50),
            Signal = 10^median(GROseq),
            SDR = mean(sdr),  SDW = mean(sdw)) %>%
  mutate(size_kb = (stop - start) / 1000) %>%  mutate(size_range = "0-300kb") %>% mutate(size_range = replace(size_range,size_kb >= 300, "300kb+")) %>%
  mutate(Timing = "Early") %>% mutate(Timing = replace(Timing,S50 > 0.5, "Late")) %>%
  mutate(Feature = "Bulk") %>% mutate(Feature = replace(Feature, SDW > 0, "SDW")) %>% mutate(Feature = replace(Feature, SDR > 0, "SDR"))
genes.reshaped_data$Timing <- factor(genes.reshaped_data$Timing,levels = c("Early","Late"))
genes.reshaped_data$size_range <- factor(genes.reshaped_data$size_range,levels = c("0-300kb","300kb+"))

### Filter Non expressed genes :
genes.reshaped_data <- subset(genes.reshaped_data,RPKM1 > 0.0001 & RPKM2 > 0.0001)

### Assemble data for plotting :
genes.bulk <- genes.reshaped_data %>% mutate(Feature_factor = "Bulk")
genes.bulk.early <- subset(genes.reshaped_data, Timing == "Early") %>% mutate(Feature_factor = "Bulk Early")
genes.long.early <- subset(genes.reshaped_data, Timing == "Early" & size_range == "300kb+") %>% mutate(Feature_factor = "300kb+ Early")
genes.bulk.late <- subset(genes.reshaped_data, Timing == "Late") %>% mutate(Feature_factor = "Bulk Late")
genes.long.late <- subset(genes.reshaped_data, Timing == "Late" & size_range == "300kb+") %>% mutate(Feature_factor = "300kb+ Late")
genes.sdr <- subset(genes.reshaped_data, Feature == "SDR") %>% mutate(Feature_factor = "SDR")
genes.sdw <- subset(genes.reshaped_data, Feature == "SDW") %>% mutate(Feature_factor = "SDW")

genes.assembled_data <- rbind(genes.bulk, genes.bulk.early, genes.long.early, genes.bulk.late, genes.long.late, genes.sdr, genes.sdw)

### Reorganise features levels order : 
genes.assembled_data$Feature_factor <- factor(genes.assembled_data$Feature_factor, levels = c("Bulk","Bulk Early","300kb+ Early","Bulk Late","300kb+ Late","SDR","SDW"))

### Write table for the plot :
write.table(genes.assembled_data,file="OUTPUTS/Plot.table.txt",row.names = FALSE, quote = FALSE,sep = "\t")
###
```

### 2.3 - Statistical tests :

```
### P-Values

test_groups <- function(x,a,b, test_name = "KS") {
  
  group_a <- subset(x,Feature_factor == a)$Signal
  group_b <- subset(x,Feature_factor == b)$Signal
  
  if (test_name == "Wilcox") {
    temp.pval <- wilcox.test(group_a,group_b)$p.value
  }
  else {
    temp.pval <- ks.test(group_a,group_b)$p.value
  }
  return(temp.pval)
}

### Bulk Vs long genes :
######
KS1 <- test_groups(genes.assembled_data,"Bulk Early","300kb+ Early")
######
KS2 <- test_groups(genes.assembled_data,"Bulk Late","300kb+ Late")
######
### Long genes vs SDR :
######
KS3 <- test_groups(genes.assembled_data,"300kb+ Early","SDR")
######
KS4 <- test_groups(genes.assembled_data,"300kb+ Late","SDR")
######
### Long genes Early vs Late :
######
KS5 <- test_groups(genes.assembled_data,"300kb+ Early","300kb+ Late")
######

temp.pval <- data.frame(Tests = c("Bulk Early Vs 300kb+ Early",
                                  "Bulk Late Vs 300kb+ Late",
                                  "300kb+ Early Vs SDR",
                                  "300kb+ Late Vs SDR",
                                  "Early Vs Late 300kb+"),
                        KS_test = c(KS1,KS2,KS3,KS4,KS5))

knitr::kable(temp.pval)
```

| Tests | KS\_test |
| --- | --- |
| Bulk Early Vs 300kb+ Early | 0.000000 |
| Bulk Late Vs 300kb+ Late | 0.000000 |
| 300kb+ Early Vs SDR | 0.016923 |
| 300kb+ Late Vs SDR | 0.000000 |
| Early Vs Late 300kb+ | 0.000000 |

# 3 - PLOT :

```
############## PLOT #############

p <- ggplot(genes.assembled_data, aes(x = Feature_factor, y = Signal, fill = Feature_factor)) +
  geom_boxplot(outlier.shape = NA, size = 1.2) +
  theme_bw() +
  scale_fill_manual(values = c("white","white","white","white","white","red","darkolivegreen4")) +
  coord_cartesian(ylim = c(0,200)) +
  geom_segment(x=2,xend=3,y=175,yend=175,size = 1) +
  geom_segment(x=4,xend=5,y=100,yend=100,size = 1) +
  geom_segment(x=5,xend=6,y=75,yend=75,size = 1) +
  geom_segment(x=3,xend=6,y=125,yend=125,size = 1) +
  geom_segment(x=3,xend=5,y=150,yend=150,size = 1) +
  annotate(geom="text",x=2.5,y=177,label="***",face="bold",size = 12) +
  annotate(geom="text",x=4.5,y=102,label="***",face="bold",size = 12) +
  annotate(geom="text",x=5.5,y=77,label="***",face="bold",size = 12) +
  annotate(geom="text",x=4.5,y=127,label="**",face="bold",size = 12) +
  annotate(geom="text",x=4,y=152,label="***",face="bold",size = 12) +
  theme(axis.text.y = element_text(size = 18, color = "black"),
        axis.text.x = element_text(size = 18, color = "black",angle = 45,vjust = 0.5),
        axis.title = element_text(size = 20),
        legend.text = element_text(size = 18), legend.position = "none",
        panel.border = element_rect(fill = NA, size = 3,color = "black")) +
  labs(x = "",y = "Expression level (RPK)",fill ="")
p
```

```
ggsave(file = "OUTPUTS/1D.png", plot = p)
```
