# Supplementary material for: Transcription-mediated organization of the replication initiation program across large genes sets common fragile sites genome-wide
Source: Nat Commun. 2019 Dec 13;10:5693. doi: 10.1038/s41467-019-13674-5 (PMC6911102; doi:10.1038/s41467-019-13674-5)
Supplement: Supplementary file 8 — Source Data [file 41467_2019_13674_MOESM8_ESM.zip › Sources_Data.NCOMMS-19-27604A/Sources_Data.Figure1.zip!/Figure1/1c/C_Mean_replicates_S50_SDR_SDW.html]

Timing S50 in SDR and SDW


# Timing S50 in SDR and SDW

#### Sami EL HILALI

#### 12 octobre 2018

- 0 - IMPORTS :
- 1 - DATA INPUT :
- 2 - DATA INTEGRATION :
- 3 - PLOT :

# 0 - IMPORTS :

```
library(dplyr)
library(ggplot2)
```

# 1 - DATA INPUT :

```
### Data paths :
SDR_path <- "../../DATA/SDRW/SDR.bed"
SDW_path <- "../../DATA/SDRW/SDW.clean.bed"
NT1_path <- "../../DATA/REPLISEQ/NT1/50kb_adj_noise_15M"
NT2_path <- "../../DATA/REPLISEQ/NT2/50kb_adj_15M"
NT3_path <- "../../DATA/REPLISEQ/NT3/50kb_adj_15M"
Aph1_path <- "../../DATA/REPLISEQ/Aph1/50kb_adj_noise_15M"
Aph2_path <- "../../DATA/REPLISEQ/Aph2/50kb_adj_noise_15M"

### Load data :

SDR <- read.csv(SDR_path,header=FALSE,sep="") %>%
  rename(chr = V1, start = V2, stop = V3)

SDW <- read.csv(SDW_path,header=FALSE,sep="") %>%
  rename(chr = V1, start = V2, stop = V3)

NT1 <- Rscripts.publi.714717::load_repliseq_df(NT1_path,"NT1")
NT2 <- Rscripts.publi.714717::load_repliseq_df(NT2_path,"NT2")
NT3 <- Rscripts.publi.714717::load_repliseq_df(NT3_path,"NT3")
Aph1 <- Rscripts.publi.714717::load_repliseq_df(Aph1_path,"Aph1")
Aph2 <- Rscripts.publi.714717::load_repliseq_df(Aph2_path,"Aph2")
```

# 2 - DATA INTEGRATION :

```
### Calculate Mean S50 for each condition :
S50 <- data.frame(chr = NT1$chr,
                  start = NT1$start,
                  stop = NT1$stop,
                  NT1_S50 = Rscripts.publi.714717::calculate_S50(NT1)$S50,
                  NT2_S50 = Rscripts.publi.714717::calculate_S50(NT2)$S50,
                  NT3_S50 = Rscripts.publi.714717::calculate_S50(NT3)$S50,
                  Aph1_S50 = Rscripts.publi.714717::calculate_S50(Aph1)$S50,
                  Aph2_S50 = Rscripts.publi.714717::calculate_S50(Aph2)$S50) %>%
  mutate(NT = (NT1_S50 + NT2_S50 + NT3_S50) / 3 ,
         Aph = (Aph1_S50 + Aph2_S50) / 2 ) %>%
  select(chr,start,stop,NT,Aph)
###
```

```
### Calculate mean S50 at SDRW positions :
Calculate_SDRW_mean_S50 <- function(regions,S50_sample,regions_name) {
  temp <- tidygenomics::genome_join_closest(regions,S50_sample)
  temp <- subset(temp,start.y >= start.x & stop.y <= stop.x) %>%
    rename(chr = chr.x,start = start.x, stop = stop.x) %>% group_by(chr,start,stop) %>%
    summarise(mean_NT = mean(NT),
              mean_Aph = mean(Aph)) %>%
    mutate(region = regions_name) %>%
    select(chr,start,stop,mean_NT,mean_Aph,region) %>%
    rename(NT = mean_NT, Aph = mean_Aph)
  return(temp)
}

### Integrate genome, SDR and SDW values : 
Bulk.S50 <- S50 %>% mutate(region = "Genome") %>% select(chr,start,stop,NT,Aph,region)
SDR.S50 <- Calculate_SDRW_mean_S50(SDR,S50,regions_name = "SDR")
SDW.S50 <- Calculate_SDRW_mean_S50(SDW,S50,regions_name = "SDW")
S50.all_integrated <- rbind.data.frame(Bulk.S50,SDR.S50)
S50.all_integrated <- rbind.data.frame(S50.all_integrated,SDW.S50) %>%
  mutate(NT = replace(NT,TRUE,round(NT,digits = 3)),
         Aph = replace(Aph,TRUE,round(Aph,digits = 3)))

### Export data table :
write.table(S50.all_integrated, file = "OUTPUTS/Mean_S50_SDRW.txt",sep="\t",quote=FALSE,row.names = FALSE)
###
```

# 3 - PLOT :

```
### Put the integrated data in histogram :
calculate_histograms_df <- function(input_df,bin,Sample) {
  
  mids <- NULL
  percents <- NULL
  regions <- NULL
  
  for (i in c("Genome","SDR","SDW")) {
    temp <- subset(input_df, region == i)
    temp.sample <- temp[[Sample]]
    temp.sample <- temp.sample[temp.sample != 0]
    h <- hist(as.numeric(temp.sample) , breaks = seq(0,1,bin), plot = FALSE)
    h$percent <- h$counts / sum(h$counts) * 100
    ##
    mids <- c(mids,h$mids)
    percents <- c(percents,h$percent)
    regions <- c(regions,rep(i,times=length(h$mids)))
  }
  output_df <- data.frame(mids = mids,
                          percents = percents,
                          regions = regions) %>%
    mutate(sample = Sample)
  output_df$sample <- factor(output_df$sample, levels = c("NT","Aph"))

  return(output_df)
  
}

NT.histogramed <- calculate_histograms_df(S50.all_integrated,bin = 0.01, Sample = "NT")
Aph.histogramed <- calculate_histograms_df(S50.all_integrated,bin = 0.01, Sample = "Aph")
All.histogramed <- rbind(NT.histogramed,Aph.histogramed)

### Write table for the plot :
write.table(All.histogramed,file="OUTPUTS/Plot.table.txt",row.names = FALSE, quote = FALSE,sep = "\t")
###

############## PLOT #############
YLIM = c(0,7.5)
# alpha = 0.25 (span) and bin = 0.01

annotation_text <- data.frame(label = c("NT","Aph"),
                              mids = c(0.125,0.125),
                              percents = c(6.5,6.5),
                              sample = c("NT","Aph"),
                              regions = c("Genome","Genome"))


p <- ggplot(All.histogramed,aes(x = mids, y = percents,color = regions)) +
  geom_smooth(span = 0.25, se = FALSE, size = 1.5) +
  scale_color_manual(values = c("black","red","darkolivegreen4")) +
  coord_cartesian(ylim = YLIM) +
  facet_grid(sample~.) +
  geom_vline(xintercept = c(0,0.25,0.5,0.75,1),linetype = "dotted") +
  theme_bw() +
  theme(legend.position = "top",legend.title = element_blank(),legend.text = element_text(size=18),
        plot.title = element_blank(),
        axis.title.y = element_text(vjust =0.5,size=22),
        axis.title.x = element_text(size=22),
        axis.text = element_text(size=18,color = "black"),
        strip.text = element_blank(),
        panel.border = element_rect(fill = NA, size = 3,color = "black")) +
  geom_text(data = annotation_text,aes(x = mids,y = percents,label = label),size = 8,show.legend = FALSE) +
  labs(x = "S50",y = "Percentage of 50 kb windows")

### Export plot as PNG :

ggsave(file = "OUTPUTS/1C.png", plot = p)

print(p)
```
