# Supplementary material for: Transcription-mediated organization of the replication initiation program across large genes sets common fragile sites genome-wide
Source: Nat Commun. 2019 Dec 13;10:5693. doi: 10.1038/s41467-019-13674-5 (PMC6911102; doi:10.1038/s41467-019-13674-5)
Supplement: Supplementary file 8 — Source Data [file 41467_2019_13674_MOESM8_ESM.zip › Sources_Data.NCOMMS-19-27604A/Sources_Data.Figure2.zip!/Figure2/2a/A_interbubbles_vs_SDRW.html]

Interbubbles in Bulk, SDR and SDW


# Interbubbles in Bulk, SDR and SDW

#### Sami EL HILALI

#### 26 novembre 2018

- 0 - IMPORTS :
- 1 - DATA INPUT :
- 2 - DATA INTEGRATION :
  - 2.1 - Calculate interbubbles :
  - 2.2 - Integrate Interbubbles with SDRW :
- 3 - PLOT :

# 0 - IMPORTS :

```
library(dplyr)
library(ggplot2)
library(scales)
```

# 1 - DATA INPUT :

This analysis features publicaly available data :  
- Bubble-seq data (GEO : GSE38809)

# 2 - DATA INTEGRATION :

### 2.1 - Calculate interbubbles :

```
# ./Calculate_Interbubbles.sh
```

### 2.2 - Integrate Interbubbles with SDRW :

```
# bedtools intersect -a OUTPUTS/Interbubbles.bed -b ../../DATA/SDRW/SDW.clean.bed -wao | awk '{if ($4 == ".") {print $1"\t"$2"\t"$3"\t"$3-$2"\tBulk"} else {print$1"\t"$2"\t"$3"\t"$3-$2"\tSDW"}}' | bedtools intersect -a - -b ../../DATA/SDRW/SDR.bed -wao | awk '{if ($6 == ".") { print $1"\t"$2"\t"$3"\t"$4"\t"$5} else {print $1"\t"$2"\t"$3"\t"$4"\tSDR"}}' > OUTPUTS/interbubbles_integrated.txt
```

# 3 - PLOT :

```
### LOAD DATA : 

interbubbles.integrated <- read.csv(file = "OUTPUTS/interbubbles_integrated.txt",sep = "",header = FALSE) %>%
  rename(chr = V1, start = V2, stop = V3, size = V4, region = V5)


interbubbles.integrated <- subset(interbubbles.integrated,size > 1)

########## reshape the data :
reshape_data <- function(x,region_i) {
  temp <- subset(x,region == region_i)
  temp_grouped <- temp %>%
    mutate(size_log = log10(size)) %>%
    group_by(size_log) %>%
    summarise(n = n()) %>%
    mutate(freq = (n / sum(n)) * 100) %>%
    mutate(region = region_i)
  return(temp_grouped)
}


### SDR
SDR <- reshape_data(interbubbles.integrated,"SDR")
#### SDW
SDW <- reshape_data(interbubbles.integrated,"SDW")
### BULK : 
Bulk <- reshape_data(interbubbles.integrated,"Bulk")
### All :
tempAll <- rbind(SDR,SDW,Bulk)
tempAll$region <- factor(tempAll$region,levels = c("Bulk","SDR","SDW"))

### Bulk
sizes.bulk <- tempAll$size_log[tempAll$region == "Bulk"]
## SDR
sizes.sdr <- tempAll$size_log[tempAll$region == "SDR"]
## SDW
sizes.sdw <- tempAll$size_log[tempAll$region == "SDW"]

######### Compute proportions histogram :
bin = 0.1
alpha = 0.25

h.bulk = hist(sizes.bulk, breaks=c(seq(0,8,bin)), plot = FALSE )
h.bulk$percent = h.bulk$counts/sum(h.bulk$counts)*100
h.SDR = hist(sizes.sdr, breaks=c(seq(0,8,bin)), plot = FALSE )
h.SDR$percent = h.SDR$counts/sum(h.SDR$counts)*100
h.SDW = hist(sizes.sdw, breaks=c(seq(0,8,bin)), plot = FALSE )
h.SDW$percent = h.SDW$counts/sum(h.SDW$counts)*100


mids <- c(h.bulk$mids,h.SDR$mids,h.SDW$mids)
percent <- c(h.bulk$percent,h.SDR$percent, h.SDW$percent)
regions <- c(rep("Bulk",times=length(h.bulk$mids)),
             rep("SDR",times=length(h.SDR$mids)),
             rep("SDW",times=length(h.SDW$percent)))

A <- data.frame(mids = mids,
                percent = percent,
                regions = regions)

### Write table for the plot
write.table(A,file="OUTPUTS/Plot.table.txt",row.names = FALSE, quote = FALSE,sep = "\t")
###

############## PLOT #############

p <- ggplot(A,aes(x = mids, y = percent, color = regions)) +
  geom_smooth(span = alpha, se = FALSE) +
  theme_bw() +
  scale_color_manual(values = c("black","red","darkolivegreen4")) +
  coord_cartesian(ylim = c(0,10)) +
  geom_vline(xintercept = c(0,2,4,6,8),linetype = "dotted") +
  theme(plot.title = element_text(face = "bold.italic",size = 12),
        axis.title.y = element_text(vjust =0.5,size=20),
        axis.title.x = element_text(size=20),
        axis.text = element_text(size=18,color ="black"),
        # axis.ticks.x = element_blank(),
        legend.title = element_blank(),
        legend.text = element_text(size=18),
        legend.position = "top",
        panel.border = element_rect(fill = NA, size = 3,color = "black")) +
  labs(x = "log10(inter init zones distances) in bp",y = "Percentage of \n inter-initiation zone distances")


### Export plot as PNG :

ggsave(file = "OUTPUTS/2A.png", plot = p)

print(p)
```
