# Supplementary material for: Transcription-mediated organization of the replication initiation program across large genes sets common fragile sites genome-wide
Source: Nat Commun. 2019 Dec 13;10:5693. doi: 10.1038/s41467-019-13674-5 (PMC6911102; doi:10.1038/s41467-019-13674-5)
Supplement: Supplementary file 8 — Source Data [file 41467_2019_13674_MOESM8_ESM.zip › Sources_Data.NCOMMS-19-27604A/Sources_Data.Figure2.zip!/Figure2/2e/E_Downslopes_near_SDR.html]

Down slope near SDR in long genes


# Down slope near SDR in long genes

#### Sami EL HILALI

#### 26 Mars 2019

- 0 - IMPORTS :
- 1 - DATA INPUTS :
- 2 - DATA INTEGRATION :
- 3 - PLOT :
  - 3.1 - Upstream & Downstream sizes :
  - 3.2 - Slopes :

# 0 - IMPORTS :

```
library(dplyr)
library(ggplot2)
```

# 1 - DATA INPUTS :

The script calculating the RAI over the downslopes take as input :  
- Selected\_SDR.genes.bed  
- RAI.NT1vsAph1.50kb.bed  
- sdr.full\_down\_slope.bed

```
### LOAD Down slopes sizes data :
down_slopes <- read.csv("../../DATA/Down_slopes/SDR.down_slopes.exact_gene_pos.strand.tab",header= FALSE, sep = "") %>%
  rename(GeneName = V1,upstream = V2, downstream = V3, gene_direction = V4, gene_size = V5)

down_slopes <- subset(down_slopes, upstream > 0 & downstream > 0)
```

# 2 - DATA INTEGRATION :

```
echo "gene_name,gene_orientation,slope_type,URI,mid_window,mid_SDR,start_SDR,stop_SDR" > OUTPUTS/Slope_SDR_gene.tab

awk '{if ($7 == "+") {print $1"\t"$2-($4*1000)"\t"$2+50000"\t"$6"\t"$7"\tupstream\t"$2"\t"$3"\n"$1"\t"$3-50000"\t"$3+($5*1000)"\t"$6"\t"$7"\tdownstream\t"$2"\t"$3} else {print $1"\t"$2-($5*1000)"\t"$2+50000"\t"$6"\t"$7"\tdownstream\t"$2"\t"$3"\n"$1"\t"$3-50000"\t"$3+($4*1000)"\t"$6"\t"$7"\tupstream\t"$2"\t"$3}}' ../../DATA/Down_slopes/SDR.down_slopes.bed | bedtools intersect -a - -b ../../DATA/Down_slopes/Selected_SDR.genes.bed | bedtools intersect -a - -b ../../DATA/URI/URI.NT1vsAph1.50kb.bed -wao | awk '{print $4","$5","$6","$12","($10+$11)/2","($7+$8)/2","$7","$8}' >> OUTPUTS/Slope_SDR_gene.tab
```

# 3 - PLOT :

There are 27 selected SDR which are hosted within long transcribed genes (see Table S1). One SDR was removed in this report compared to the previous relative reports, as it was overlapping the extremitie of the long transcribed gene, a part of it being outside the gene. This SDR was located at gene MBD5.

### 3.1 - Upstream & Downstream sizes :

```
############## PLOT #############

p <- ggplot(down_slopes,aes(x= upstream, y = downstream,color = gene_direction)) +
  geom_jitter(aes(size = gene_size)) +
  theme_bw() +
  geom_abline(slope = 1, intercept = 0, linetype = "dashed") +
  coord_cartesian(xlim = c(0,300),ylim = c(0,300)) +
  scale_color_manual(values = c("blue2", "deepskyblue")) +
  theme(axis.title = element_text(size = 22),
        legend.position = "top",
        axis.text = element_text(size = 18,color = "black"),
        legend.text = element_text(size = 18, color = "black"),
        legend.title = element_text(size = 18, color = "black"),
        plot.title = element_text(size = 15,face = "bold" ),
        panel.border = element_rect(fill = NA, size = 3,color = "black")) +
labs(x= "Upstream size (kb)", y = "Downstream size (kb)",color = "Gene orientation",size = "size :")

### Export plot as PNG :

ggsave(file = "OUTPUTS/2D_top.png", plot = p)

print(p)
```

### 3.2 - Slopes :

```
### LOAD Down slopes  RAI data :
slope.gene <- read.csv("OUTPUTS/Slope_SDR_gene.tab") %>%
  group_by(gene_name,slope_type) %>%
  mutate(mid_window_scaled = (mid_window - mid_SDR)/1000) %>%
  mutate(start_window_scaled = (mid_window - start_SDR)/1000) %>%
  mutate(start_window_scaled = replace(start_window_scaled,gene_orientation == "+" && slope_type == "downstream",(mid_window - stop_SDR + 25000)/1000)) %>%
  mutate(start_window_scaled = replace(start_window_scaled,gene_orientation == "+" && slope_type == "upstream",(mid_window - start_SDR - 25000)/1000)) %>%
  mutate(start_window_scaled = replace(start_window_scaled,gene_orientation == "-" && slope_type == "downstream",(mid_window - start_SDR - 25000)/1000)) %>%
  mutate(start_window_scaled = replace(start_window_scaled,gene_orientation == "-" && slope_type == "upstream",(mid_window - stop_SDR + 25000)/1000))
slope.gene <- subset(slope.gene,gene_name != "MBD5")

############## PLOT #############
p <- ggplot(slope.gene,aes(x= start_window_scaled,y = URI)) +
  geom_line(aes(color = gene_name,linetype = slope_type)) +
  coord_cartesian(ylim = c(-3,2.5)) +
  theme_bw() +
  geom_vline(xintercept = 0,linetype = "dotted") +
  geom_hline(yintercept = -2, linetype = "dashed") +
  theme(axis.title = element_text(size = 22),
        legend.position = "none",
        axis.text = element_text(size = 18,color = "black"),
        legend.text = element_text(size = 18, color = "black"),
        legend.title = element_text(size = 18, color = "black"),
        plot.title = element_text(size = 15,face = "bold" ),
        panel.border = element_rect(fill = NA, size = 3,color = "black")) +
  labs(x="Position relative to T-SDR (kb)")


### Export plot as PNG :

ggsave(file = "OUTPUTS/2D_bottom.png", plot = p)

print(p)
```
